# Exercise intensity determines circulating levels of Lac-Phe and other exerkines: a randomized crossover trial

**DOI:** 10.1007/s11306-025-02260-0

**Published:** 2025-05-07

**Authors:** Dirk Weber, Paola G. Ferrario, Achim Bub

**Affiliations:** 1 Institute of Sports and Sports Science, Karlsruhe Institute of Technology (KIT), Engler-Bunte-Ring 15, 76131 Karlsruhe, Germany; 2 Department of Physiology and Biochemistry of Nutrition, Max Rubner-Institute, Karlsruhe, Germany

**Keywords:** Exercise metabolomics, Exerkine, Exercise intensity, N-lactoyl-phenylalanine (Lac-Phe), N-acetylated amino acids, Acylcholines

## Abstract

**Introduction:**

Exercise metabolomics research has revealed significant exercise-induced metabolic changes and identified several exerkines as mediators of physiological adaptations to exercise. However, the effect of exercise intensity on metabolic changes and circulating exerkine levels remains to be examined.

**Objectives:**

This study compared the metabolic responses to moderate-intensity and vigorous-intensity aerobic exercise.

**Methods:**

A two-period crossover trial was conducted under controlled conditions at the Max Rubner-Institute in Karlsruhe, Germany. Seventeen young, healthy, and physically active men performed 30 min moderate-intensity (50% VO_2peak_) and vigorous-intensity (75% VO_2peak_) aerobic exercise using two bicycle ergometer protocols in a randomized sequence. Blood samples obtained immediately before exercise and at four time points after exercise were analyzed in an untargeted metabolomics approach, and separate linear mixed models were applied to over 1000 metabolites.

**Results:**

Vigorous-intensity exercise induced a greater metabolic response than moderate-intensity exercise. Several intensity-dependent metabolites were identified, primarily involved in amino acid metabolism and energy conversion pathways, including N-lactoyl-amino acids, TCA cycle intermediates, N-acetylated amino acids, and acylcholines. The exerkines N-lactoyl-phenylalanine, lactate, and succinate were among the most intensity-dependent metabolites. N-acetylated amino acids and acylcholines were systematically altered by exercise intensity, indicating potential physiological functions.

**Conclusion:**

Exercise intensity significantly affects exercise-induced metabolic alterations and changes in exerkine levels. Our results expand the knowledge about exerkine dynamics and emphasize the role of exercise intensity in promoting physiological adaptations to exercise.

The trial was registered on October 5, 2017, at the German Clinical Trials Register under the Registration Number DRKS00009743 (Universal Trial Number of WHO: U1111-1200-2530).

**Supplementary Information:**

The online version contains supplementary material available at 10.1007/s11306-025-02260-0.

## Introduction

Aerobic exercise provides a wide range of health benefits, including significant reduction in the risk of cardiovascular diseases, type 2 diabetes mellitus, and all-cause mortality (Posadzki et al., 2020; Wahid et al., 2016). Nevertheless, the molecular mechanisms underlying the beneficial health outcomes associated with exercise remain insufficiently understood (Sanford et al., 2020). In recent years, metabolomics has emerged as a powerful approach to investigate the molecular responses to sports and exercise (Khoramipour et al., 2022). Untargeted metabolomics studies have provided a comprehensive overview of the metabolic responses to physical activity, enabling the characterization of metabolic signatures across different populations and exercise modalities, the profiling of health- and disease-related metabolic states, and the identification of novel exercise-induced pathways and metabolites (Kelly et al., 2020; Schranner et al., 2020). As such, studies have identified several exercise-inducible signaling molecules, termed exerkines, which mediate physiological responses to exercise and may contribute to health benefits and disease prevention (Chow et al., 2022).

Exercise-induced metabolic alterations primarily involve key pathways, including lipid metabolism, amino acid metabolism, glycolysis, and the tricarboxylic acid (TCA) cycle (Kelly et al., 2020; Schranner et al., 2020). Intermediates of these pathways, such as lactate and succinate, have been identified as exerkines with diverse physiological functions, including signaling tissue remodeling and metabolic adaptations (Brooks et al., 2022; Mills et al., 2018; Reddy et al., 2020). Recently, N-lactoyl-phenylalanine (Lac-Phe), which is synthesized from lactate and phenylalanine, by the cytosolic enzyme CNDP2, has been identified as an exerkine that suppresses post-exercise hunger and food intake (Jansen et al., 2015; Li et al., 2022). Lac-Phe decreased food intake of diet-induced obese mice without affecting ambulatory activity or energy expenditure, and in humans, Lac-Phe was one of the most exercise-regulated metabolites, showing elevated levels for up to 3 h after exercise (Li et al., 2022). Additionally, recent research suggests that Lac-Phe mediates the anti-obesity effects of Metformin, further emphasizing its therapeutic potential (Scott et al., 2024; Xiao et al., 2024).

However, exercise metabolomics studies vary considerably in their objectives and methods and cover a wide range of sports and exercise modalities. Based on data from a human exercise metabolomics study with varying exercise protocols, Li et al. concluded that distinct exercise modalities lead to different post-exercise Lac-Phe levels (Li et al., 2022). Nevertheless, the specific exercise modalities required for substantial synthesis of Lac-Phe and other exerkines remain incompletely understood. Previous research indicates that variations in exercise intensity may markedly influence metabolic changes (Kelly et al., 2020; Khoramipour et al., 2022). However, the effect of exercise intensity on metabolic alterations and circulating exerkine levels has not yet been systematically investigated.

To address this gap, the present study compared the metabolic responses of young, healthy, and physically active men after 30 min of continuous moderate-intensity aerobic exercise (CME) and 30 min of continuous vigorous-intensity aerobic exercise (CVE). We hypothesized that vigorous-intensity exercise would lead to more pronounced metabolic changes than moderate-intensity exercise. In particular, we aimed to determine whether circulating levels of exerkines such as Lac-Phe change in an intensity-dependent manner.

## Methods

### Study participants and anthropometry

Young, healthy, and physically active men aged 18 to 30 (*n* = 17) were recruited for this acute exercise trial conducted at the Max Rubner-Institute in Karlsruhe, Germany, between October 2017 and May 2018. All inclusion and exclusion criteria are given in Supplementary Table 1. Before study onset, body weight and height were measured with the participants in underwear and without shoes, and body mass index was calculated. Bioelectrical impedance analysis was used to assess body composition, and lean body mass, fat mass, and percent fat mass were calculated. Additionally, resting heart rate (HR_rest_) and systolic and diastolic blood pressure were measured after a resting period of 5 min in a sitting position. All instruments used in this study, along with their respective applications, are detailed in Supplementary Table 2. A more comprehensive description of the study design has been reported elsewhere (Kistner et al., 2023).

The sample size calculation was performed for the primary outcome of the study. A total of 17 participants were required to achieve 80% power at a 5% significance level, accounting for a 20% drop-out rate, under the crossover design. The sample size calculation was made using the R package TrialSize (function TwoSampleCrossOver.Equality, version 3.3.2). The metabolomics analysis was explorative.

### Experimental design

To establish individualized protocols for the acute exercise trials, participants visited the study center on a separate day before study onset, performing a cardiopulmonary exercise test on a bicycle ergometer. The peak oxygen uptake (VO_2peak_) was assessed to determine the workload of the experimental trials. For each participant, the workload was calculated as 50% (CME trial) and 75% (CVE trial) of the individual power at VO_2peak_. To address the inter-individual variability in the metabolome of participants, a two-period crossover study design was used. Each participant completed both CME and CVE trials in a randomized sequence, according to a computer-generated allocation schedule based on simple randomization. Participants were enrolled by the study recruitment staff, and assigned to the sequence of interventions by the study organization. In total, 10 participants were allocated to perform the CVE trial first, and 7 participants were allocated to perform the CME trial first. Since notable long-term metabolic alterations are not expected in response to a single training session (Kelly et al., 2020) a washout period of at least 1 week was deemed sufficient to minimize any potential carry-over effects. Participants were instructed to avoid intense exercise on the days preceding both exercise trials. Additionally, participants were required to adhere to a standardized diet on both exercise trial days and on the day preceding each trial. Details on the energy and macronutrient intake at breakfast and on the preceding days and water consumption during the experimental trials are available in Supplementary Table 3. For a detailed description of the preliminary exercise testing and the design of dietary plans, please refer to Kistner et al. (Kistner et al., 2023).

**Fig. 1:**
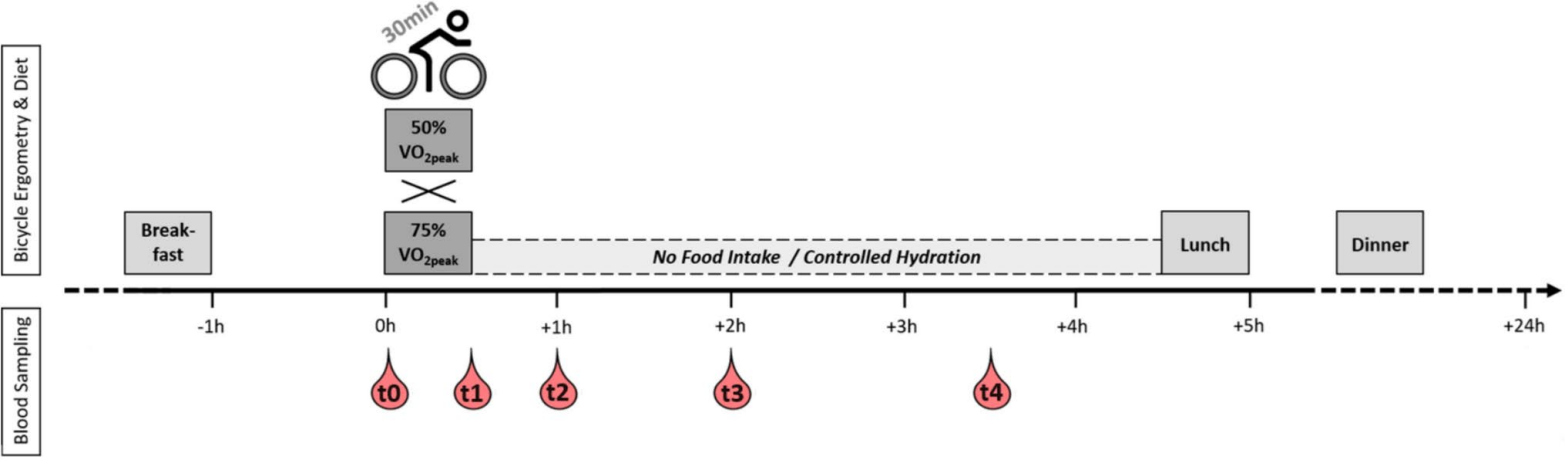
Experimental design of the study. 17 healthy, young, and physically active men completed a 30 min continuous moderate exercise (CME) trial and a 30 min continuous vigorous exercise (CVE) trial in a randomized sequence. Participants were provided with a standardized breakfast and a standardized lunch on the intervention days. Five venous blood samples were obtained for each trial. The first sample was taken at baseline, immediately before the start of the experimental trial (t_0_). The other four samples were taken immediately after exercise (t_1_) and 30 min (t_2_), 90 min (t_3_), and 180 min (t_4_) post exercise

Participants arrived at the study center at 7:30 am on both study days, following an overnight fast. Immediately after their arrival, the experimental protocol was explained to the participants, and a catheter for venous access was placed. At 90 min before the start of the experimental trial, participants were provided with a standardized breakfast meal. After consuming the breakfast, the participants rested for 1 h until the experimental trial began. Both exercise interventions were conducted between 9:00 am and 10:00 am. During the whole experimental trial, standardized environmental conditions were maintained in the testing room according to the Practice Guidelines for Exercise Testing (Wonisch et al., 2014). Immediately before the start of the trial, a first venous blood sample (t_0_) was obtained. The exercise trial started with a 2 min warm-up at 50W (CME trial) or 100W (CVE trial) followed by 30 min of continuous cycling on a bicycle ergometer at the power of either 50% VO_2peak_ (CME trial) or 75% VO_2peak_ (CVE trial). After completion of the exercise protocols, participants continued cycling at either 50W for 3 min (CME trial) or at 100W for 1 min and 50W for 2 min (CVE trial). Venous blood samples were taken from each participant immediately after exercise (t_1_) and 30 min (t_2_), 90 min (t_3_), and 180 min (t_4_) post exercise. At 240 min post exercise, participants were provided with a standardized lunch. Between the standardized breakfast and lunch, participants were mainly sitting and did not consume any food or drinks except for water. The water intake of participants was recorded, and heart rate (HR) was continuously monitored from the start of the experimental trial until lunch. Figure 1 shows the experimental design of the study. This study was conducted in accordance with the CONSORT extension for reporting randomized crossover trials (Dwan et al., 2019).

### Sample preparation and metabolomics analyses

Blood samples were collected from the catheter using S-Monovette® tubes and immediately placed on ice. The samples were centrifuged at 2500×*g* for 10 min at 4 °C to separate the plasma. Using a pipette, the plasma was carefully transferred to a sterile 15 mL tube. All plasma samples were then aliquoted into cryovials and cryopreserved in liquid nitrogen at − 196 °C. Plasma samples were sent to Metabolon (Metabolon, Inc. North Carolina, USA) for untargeted metabolomics analyses and were preserved at − 80 °C until analysis. The experimental procedure of the Metabolon platform is described in detail elsewhere (DeHaven et al., 2010; Evans et al., 2009). In brief, the samples were subdivided into multiple fractions: two were subjected to two separate reverse-phase (RP)/UPLC-MS/MS analyses with positive ion mode electrospray ionization (ESI), one was analyzed by RP/UPLC-MS/MS with negative ion mode ESI, and another was processed using HILIC/UPLC-MS/MS with negative ion mode ESI. Data processed by Metabolon led to the identification of 1262 metabolites. All metabolites of known identity were assigned to super-pathways and sub-pathways according to Metabolon’s classification, as shown in Supplementary Table 4.

### Statistical analyses

We received the unprocessed, raw peak area data from Metabolon, which displayed metabolites on a wide scale and contained missing values. To obtain a complete dataset for subsequent analyses, we imputed missing values using a random forest imputation algorithm, which has been shown to perform well in MS-based metabolomics data (Kokla et al., 2019; Wei et al., 2018). Metabolites with more than 20% missing values were excluded before imputation. This so-called 80% rule limits the potential uncertainty introduced by the imputation process (Bijlsma et al., 2006). We examined whether the distribution of missing values within the excluded metabolites differed by exercise intensity but found no consistent pattern indicative of intensity-related bias. Consequently, 237 of the initial 1262 metabolites were excluded, resulting in a final dataset of 1025 metabolites, of which 870 have been structurally identified and 155 are of unknown structural identity.

To investigate intensity-dependent metabolic changes while accounting for the crossover design, linear mixed models were used. For each metabolite, a separate model was applied with the metabolite as the dependent variable. *Time* and *Intensity* as well as their interaction were included as fixed effects along with *Participant* as a random effect to account for inter-individual variability. *Sequence* and *Period* of intervention were considered as covariates and tested in regression models of 25 randomly chosen metabolites. We compared these models using the Anova function from the base R package but found no model improvements and chose not to include these variables to reduce model complexity. After visual assessment of QQ plots of the residuals from the regression models, the assumption of normal distribution did not appear to be justified for some metabolites. Log_2_-transformation of all metabolite data allowed the use of parametric tests. QQ plots for the same 25 randomly chosen metabolites, both before and after the log₂-transformation, are provided in Supplementary Fig. 1. The interaction effect between *Time* and *Intensity* at each post-intervention time point was examined to investigate the effect of exercise intensity at specific time points in comparison with baseline. The *p*-values for each metabolite and statistical test were adjusted using the Benjamini–Hochberg procedure (Benjamini & Hochberg, 1995) to control for the false discovery rate (FDR), resulting in *q*-values. Statistical significance was defined as a *q*-value < 0.05. Additionally, we tested whether participant characteristics with high coefficients of variation, such as percentual fat mass (CV = 30.9%), resting heart rate (CV = 24.7%), power at the lactate threshold (CV = 18.9%), and power at the individual anaerobic threshold (CV = 16.2%), affected the association between *Time*, *Intensity*, and metabolite levels. Each variable was included as a three-way interaction (*Time*:*Intensity*:*Variable*) in the linear mixed models. None of these interactions were statistically significant, indicating that they did not moderate the observed exercise-related metabolic responses. Pathway enrichment analysis and calculation of odds ratios (OR) were performed using Fisher’s exact test. The resulting *p*-values were adjusted for multiple testing using the Benjamini–Hochberg procedure, with pathways considered significant at an adjusted *p*-value of < 0.05. Additionally, paired Wilcoxon signed-rank tests were used to test for differences in energy consumption, nutrient and liquid intake, and exercise parameters between the CME and CVE trials. All statistical analyses and data visualization were performed using R (Version 4.4.1).

## Results

### Participant characteristics

Table 1 displays the descriptive characteristics of the 17 healthy male participants and their exercise parameters. Participants were classified as having normal weight, as demonstrated by a mean body mass index (BMI) of 22.8. Preliminary exercise testing showed that participants had an average power at the lactate threshold (P_LT_) of 164.6W and at the individual anaerobic threshold (P_IAT_) of 215.6W. A paired Wilcoxon signed-rank test revealed that the CVE trial was performed at a significantly higher power (224.4W) than the CME trial (129.4W; *p* < 0.001). Similarly, maximum heart rate (HR_max_) was significantly higher in the CVE trial (169 bpm) than in the CME trial (132 bpm; *p* < 0.001). Additionally, we tested for differences in energy, nutrient, and water consumption between the CME and CVE trials (Supplementary Table 3). Analyses showed no significant difference for energy intake between the trials. Nevertheless, protein intake (*p* < 0.001), post-exercise water consumption (*p* < 0.001), and the percentage of energy intake from carbohydrates (*p* = 0.03) significantly differed between the exercise trials.

**Table 1:** Descriptive characteristics of participants (*n* = 17) and exercise parameters

Participant characteristics	Mean ± SD	Minimum	Maximum
Age (years)	24.8 ± 2.7	18.7	30.3
Body height (cm)	182.5 ± 5.7	174.7	191.2
Body weight (kg)	75.8 ± 7.1	67.0	90.8
Body mass index (kg m^−2^)	22.8 ± 1.9	20.2	25.9
Lean body mass (kg)	64.4 ± 6.1	56.8	75.4
Fat mass (%)	14.9 ± 4.6	7.4	24.8
Systolic blood pressure (mmHg)	124.7 ± 10.0	108.0	146.0
Diastolic blood pressure (mmHg)	76.2 ± 6.5	66.0	87.0
HR_rest_ (bpm)	62.8 ± 15.5	49.0	112.0
Preliminary exercise testing			
VO_2peak_ (mL kg^−1^ min^−1^)	56.6 ± 6.3	45.0	68.0
P_max_ (W)	340.9 ± 43.3	273.7	450.0
P_LT_ (W)	164.6 ± 31.1	113.3	238.0
P_IAT_ (W)	215.6 ± 35.0	149.5	289.6
HR_max_ (bpm)	191.3 ± 8.1	173.0	204.0
Exercise interventions			
CME trial			
P_50%_ (W)	129.4 ± 20.4	77.0	156.0
HR_max_ (bpm)	132.0 ± 13.4	109.0	153.0
CVE trial			
P_75%_ (W)	224.4 ± 31.2	163.0	270.0
HR_max_ (bpm)	169.3 ± 13.4	148.0	189.0

*SD* standard deviation, *HR*_*rest*_ resting heart rate, *VO*_*2peak*_ peak oxygen uptake, *P*_*max*_ maximum power, *P*_*LT*_ power at the lactate threshold, *P*_*IAT*_ power at the individual anaerobic threshold, *HR*_*max*_ maximum heart rate, *P*_*50%*_ power at 50% VO_2peak_, *P*_*75%*_ power at 75% VO_2peak_

### Intensity-dependent metabolic alterations

Table 2 presents the number of metabolites (total and in each super-pathway) with significant differences in post-exercise levels between CME and CVE (intensity-dependent metabolites) as well as the direction of change with increasing exercise intensity. Results were derived from the interaction effects between *Intensity* and *Time* at each of the four post-exercise time points (t_1_–t_4_) compared with baseline (t_0_) in linear mixed models. As illustrated, several intensity-dependent metabolites were identified and most of them were associated with the amino acid super-pathway. Notably, more metabolites showed increased rather than decreased levels with increasing exercise intensity. The most pronounced effects were observed immediately post exercise (85 intensity-dependent metabolites), followed by 30 min post exercise (26 intensity-dependent metabolites). A comprehensive table of all significant intensity-dependent metabolites for all post-exercise time points is provided in Supplementary Table 5. The table shows that all intensity-dependent metabolites identified at any post-exercise time point were also identified at the first post-exercise time point.

**Table 2:** Number, direction of change, and corresponding super-pathways of metabolites showing significant intensity-dependent changes at four post-exercise time points compared to baseline

Super-pathway	t_1_	t_2_	t_3_	t_4_
Significant metabolites in total	+72/−13	+22/−4	+3/−0	+2/−0
Amino Acid	+36/−2	+17/−0	+3/−0	+1/−0
Energy	+5/−0	+2/−0	+0/−0	+0/−0
Lipid	+14/−10	+1/−4	+0/−0	+0/−0
Carbohydrate	+2/−0	+0/−0	+0/−0	+0/−0
Nucleotide	+5/−0	+0/−0	+0/−0	+0/−0
Cofactors and Vitamins	+1/−0	+0/−0	+0/−0	+0/−0
Peptide	+0/−0	+0/−0	+0/−0	+0/−0
Xenobiotics	+0/−1	+0/−0	+0/−0	+0/−0
Uncharacterized	+9/−0	+2/−0	+0/−0	+1/−0

*t*_*1*_ immediately after exercise, *t*_*2*_ 30 min after exercise, *t*_*3*_ 90 min after exercise, *t*_*4*_ 180 min after exercise, *+* increasing with increasing exercise intensity; *−* decreasing with increasing exercise intensity

**Fig. 2:**
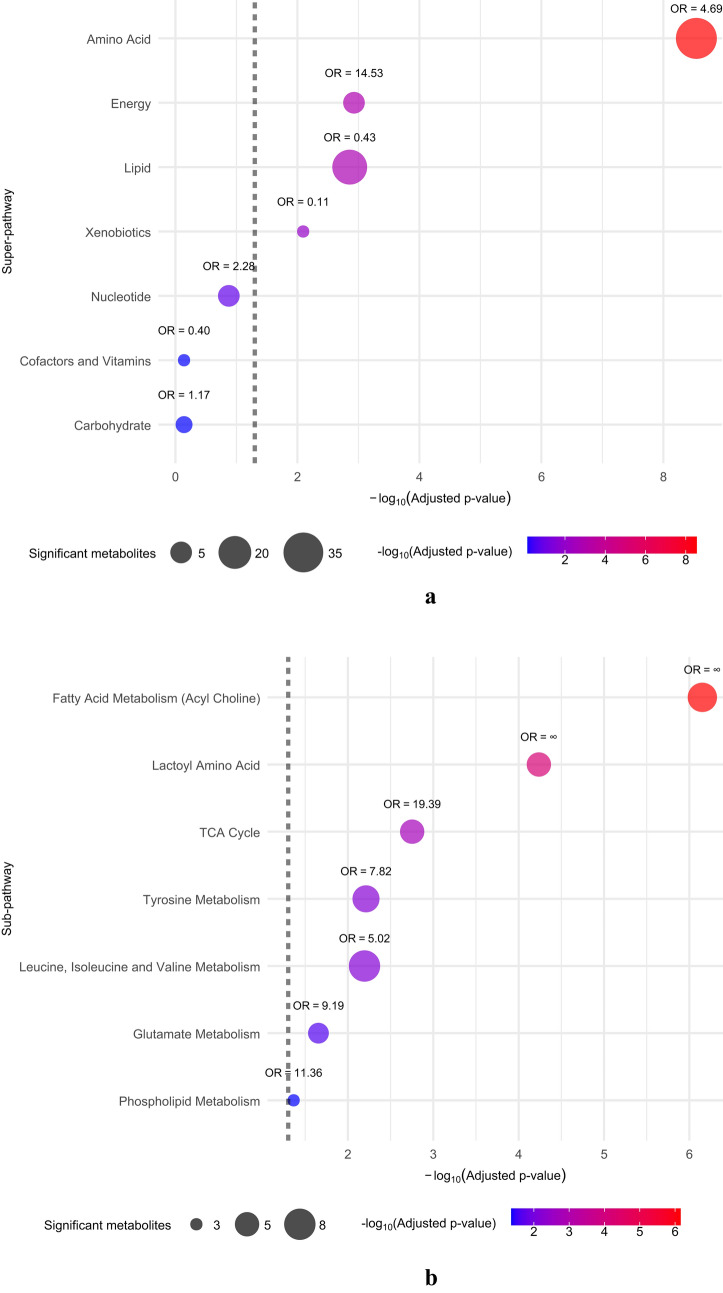
Results of the super-pathway (**a**) and sub-pathway (**b**) enrichment analyses. Fisher’s exact test was used to identify significantly enriched pathways based on Metabolon’s super-pathway and sub-pathway classification. Resulting *p*-values were adjusted for multiple testing using the Benjamini–Hochberg procedure. The grey dashed vertical line marks the significance threshold at an adjusted *p*-value of 0.05, with pathways to the right of this line considered significantly enriched. The color scale ranges from blue to red, indicating the − log_10_ of the adjusted *p*-value, with blue representing lower values and red representing higher values. Odds ratios (OR) indicate whether a pathway is overrepresented (OR > 1) or underrepresented (OR < 1) in the set of significant intensity-dependent metabolites. “OR = ∞” indicates that all analyzed metabolites from that pathway are significant. For the sub-pathway analysis, only significant sub-pathways are displayed (Color figure online)

To identify intensity-dependent pathways, super-pathway and sub-pathway enrichment analyses were performed, as shown in Fig. 2a and b, respectively. Because all intensity-dependent metabolites were observed immediately post exercise, the analyses focused on the first post-exercise time point. Pathway enrichment analysis identifies metabolic pathways that are significantly overrepresented (OR > 1) or underrepresented (OR < 1) by comparing the distribution of significant metabolites across pathways, accounting for the size of each pathway. Super-pathway enrichment analysis identified amino acid metabolism and energy metabolism as the most intensity-dependent super-pathways. Notably, the lipid and xenobiotics pathways were significant because of the markedly low number of intensity-dependent metabolites in relation to all metabolites in these pathways. The most intensity-dependent sub-pathways were lactoyl-amino acid metabolism and acylcholine metabolism. The associated OR were infinite, because all analyzed metabolites in these pathways were significantly intensity dependent. Additionally, the TCA cycle; metabolism of amino acids such as tyrosine, glutamate, and branched-chain amino acids (BCAAs); and phospholipid metabolism were found to be intensity-dependent sub-pathways.

**Fig. 3:**
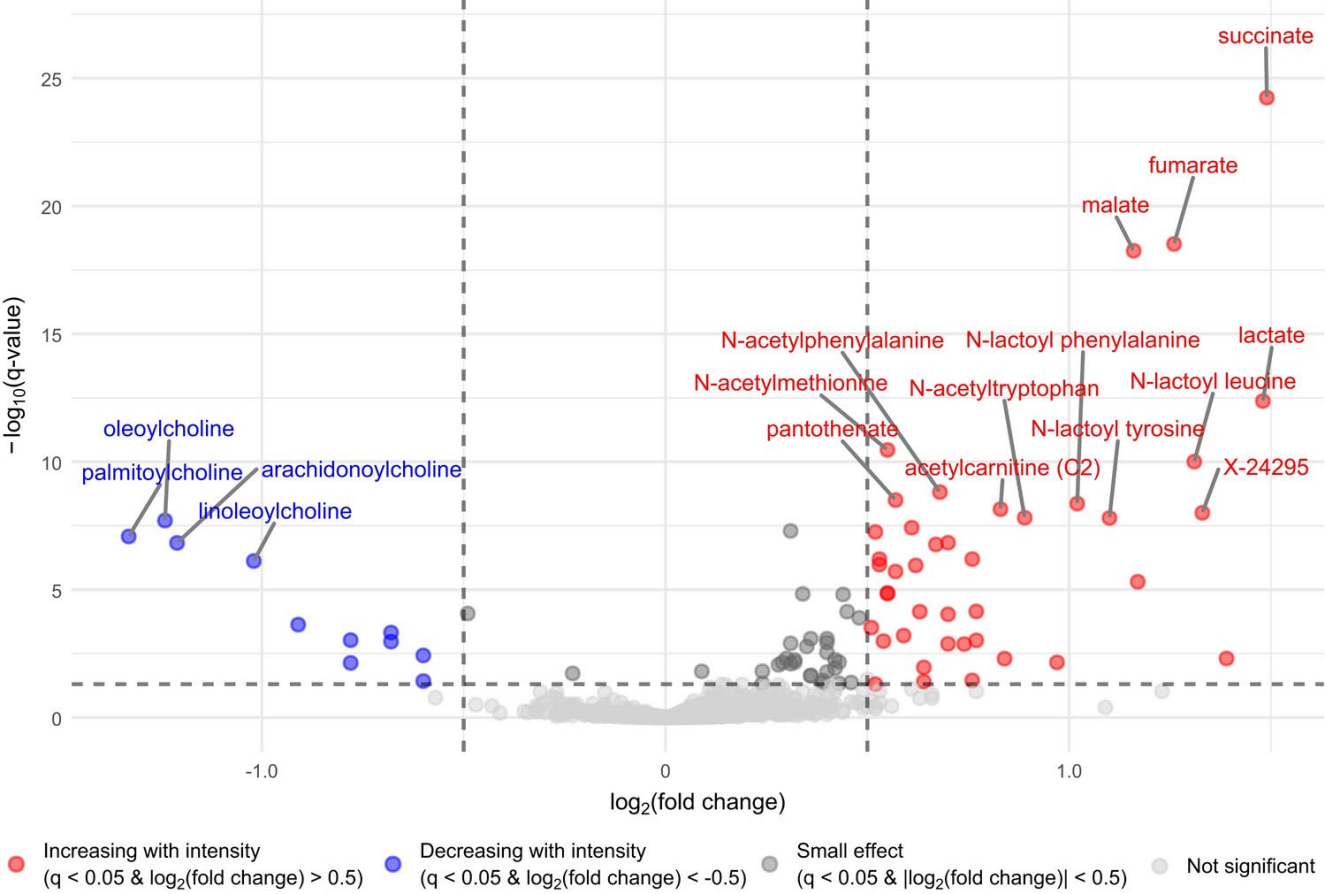
Volcano plot of metabolites immediately post exercise. The x-axis displays the log_2_ of the fold change in metabolite levels from before to immediately after exercise, based on the interaction between *Time* and *Intensity* in the mixed linear model. The y-axis represents the − log_10_ of the *q*-value. Significant intensity-dependent metabolites (*q*-value < 0.05) with log_2_(fold change) > 0.5 are shown in red. Significant intensity-dependent metabolites with log_2_(fold change) < -0.5 are shown in blue. Significant intensity-dependent metabolites but with a small effect (log_2_(|fold change|) < 0.5) are shown in dark grey and metabolites not significantly altered by exercise intensity are shown in light grey. The grey dashed lines indicate the significance thresholds for -log_10_(*q*-value) and the chosen thresholds for log_2_(fold change), respectively (Color figure online)

To illustrate the most intensity-dependent metabolites at the first post-exercise time point a volcano plot was used, as shown in Fig. 3. The results indicated that distinct metabolite groups dominate among the most intensity-dependent metabolites immediately post exercise. Intermediates of the TCA cycle, such as succinate (log_2_(fold change) = 1.49, CI [1.27, 1.7], *q*-value < 0.0001), fumarate, and malate were the most intensity-dependent metabolites. Notably, N-lactoyl-amino acids such as Lac-Phe (log_2_(fold change) = 1.02, CI [0.74, 1.31], *q*-value < 0.0001), as well as lactate (log_2_(fold change) = 1.48, CI [1.15, 1.8], *q*-value < 0.0001), were among the most intensity-dependent metabolites. Additionally, compounds with an acetyl group, such as acetylcarnitine and N-acetylated amino acids (Ac-AA) such as N-acetylphenylalanine (log_2_(fold change) = 0.68, CI [0.5, 0.86], *q*-value < 0.0001), were significantly intensity dependent. The cluster of long-chain acylcholines such as palmitoylcholine (log_2_(fold change) = − 1.33, CI [− 1.73, − 0.92], *q*-value < 0.0001), were identified as the most intensity-dependent metabolites among those that decreased with increasing exercise intensity.

**Fig. 4:**
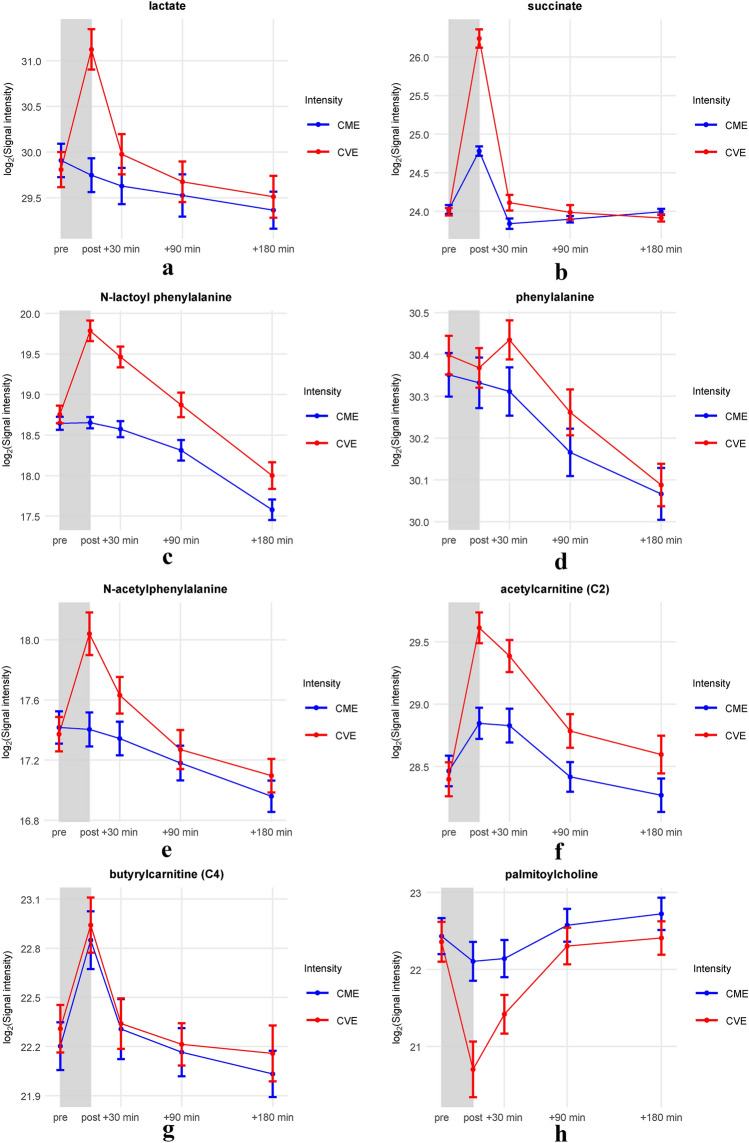
Line plots of selected metabolites. The line plots display log_2_ of the signal intensities of metabolites on the y-axis and time points (pre- and post-exercise) on the x-axis. The blue and red colors represent continuous moderate exercise (CME) and continuous vigorous exercise (CVE) trials, respectively. Bars represent the standard error of the mean (SEM) (Color figure online)

To provide a clear visualization of trends and changes over time across the intensity levels, we used line plots to represent selected metabolites (Fig. 4a–h). Visual inspection of the line plots suggests that levels of N-lactoyl-amino acids such as Lac-Phe (Fig. 4c) remained stable in response to the CME trial while peaking immediately post exercise in the CVE trial and gradually decreasing thereafter. Similarly, lactate levels (Fig. 4a) did not increase in response to the CME trial but peaked immediately post exercise in the CVE trial and decreased more rapidly to near-baseline levels within 30 min post exercise. Levels of corresponding amino acids such as phenylalanine (Fig. 4d) displayed no significant intensity dependence. To gain a better understanding of the relationship between N-lactoyl-amino acids and lactate as well as corresponding amino acids, we performed simple linear regressions on the fold change between the pre-exercise and immediately post-exercise signal intensities in the CVE trial (Supplementary Fig. 2). Notably, significant strong positive correlations were observed between the fold changes of all five N-lactoyl-amino acids and lactate, respectively. In contrast, the fold changes of the N-lactoyl-amino acids showed no correlation with those of their corresponding amino acids, except for N-lactoyl-isoleucine, which showed a moderate correlation with isoleucine. Succinate (Fig. 4b) peaked in both trials immediately post exercise and rapidly decreased thereafter but the peak is more pronounced after the CVE trial. Ac-AA such as N-acetylphenylalanine (Fig. 4e) peaked immediately post exercise in the CVE trial and decreased gradually thereafter, while no peak can be observed in response to the CME trial. A similar pattern was also observed in acetylcarnitine (Fig. 4f), which showed an intensity-dependent peak immediately post exercise. In contrast, other acylcarnitines such as butyrylcarnitine (Fig. 4g) peaked immediately post exercise but displayed no significant difference between intensity levels. All analyzed acylcholines such as palmitoylcholine (Fig. 4h) systematically decreased in the CVE trial and gradually increased thereafter.

## Discussion

We identified numerous significant intensity-dependent metabolites, primarily related to amino acid metabolism and energy conversion pathways. The most intensity-dependent groups of metabolites were N-lactoyl-amino acids, TCA cycle intermediates, Ac-AA and acylcholines. All significant intensity-dependent metabolites were observed immediately post exercise and most of them decreased rapidly in the post-exercise period, showing no significant difference between exercise intensities at 90 min post exercise and beyond. This is in line with previous research demonstrating a rapid return to baseline for most metabolites in the post-exercise state (Contrepois et al., 2020; Schranner et al., 2020). Moreover, the majority of intensity-dependent metabolites showed increased levels with increasing exercise intensity. Our results indicate that vigorous-intensity exercise induces a greater metabolic response than moderate-intensity exercise and highlight the role of exercise intensity in shaping metabolic responses.

We demonstrated that the levels of succinate, lactate, and Lac-Phe increase in response to exercise in an intensity-dependent manner. Notably, succinate was the most intensity-dependent metabolite and all five analyzed N-lactoyl-amino acids were among the most intensity-dependent metabolites immediately post exercise. In line with previous research we demonstrated that post-exercise accumulation of circulating N-lactoyl-amino acids is correlated with extracellular lactate levels but not with levels of corresponding amino acids (Jansen et al., 2015; Li et al., 2022). Interestingly, we observed a correlation between isoleucine and N-lactoyl-isoleucine, a finding that may be relevant given prior associations of isoleucine with metabolic health (Green et al., 2023; Yu et al., 2021). However, the current data are insufficient to draw meaningful conclusions about this correlation. Exercise intensities that lead to substantial lactate accumulation such as those above the lactate threshold (LT) or the individual anaerobic threshold (IAT) may be required for synthesis of N-lactoyl-amino acids. Among the 17 participants in the present study, the average power at both the LT (164.6W) and the IAT (215.6W), falls between the average power in the CME trial (129.4W) and the CVE trial (224.4W). As a result, the levels of both lactate and N-lactoyl-amino acids did not increase in response to the CME trial but did notably increase in response to the CVE trial. Consistent with this finding, Li et al. reported that a short but intense sprint intervention on a bicycle ergometer induced a more pronounced increase in Lac-Phe concentrations than a 90 min treadmill endurance intervention at 55% VO_2peak_. They concluded that distinct exercise modalities lead to different post-exercise Lac-Phe levels (Li et al., 2022). Our results indicate that exercise intensity is the fundamental determinant of post-exercise levels of Lac-Phe and other exerkines, and that moderate-intensity exercise may not provide a sufficient stimulus for substantial Lac-Phe accumulation.

Similarly, nine Ac-AA were identified as significantly intensity-dependent, systematically increasing with exercise intensity. The only established source of a wide range of Ac-AA is N-terminal protein acetylation, which is performed by specific N-terminal-acetyltransferases (NatA–NatF) (Drazic et al., 2016). Most Ac-AA observed to be intensity dependent in the present study are exclusively acetylated by NatC–NatF, which target only a small fraction of human proteins (Ree et al., 2018). Additionally, some of the more commonly acetylated Ac-AA, such as alanine, were not significantly altered by exercise intensity. Moreover, attributing the intensity-dependent increase in Ac-AA to protein acetylation suggests a notably enhanced muscle protein breakdown during exercise, which has not been conclusively demonstrated (Kumar et al., 2009; Rose & Richter, 2009). Recent studies indicate that non-enzymatic acetylation also occurs and depends on the availability of acetyl-CoA (Narita et al., 2019). An involvement of amino acids in acetyl-CoA metabolism could help balance acetyl-CoA levels, thereby increasing the efficiency of energy metabolism. For instance, l-carnitine/acetylcarnitine is involved in acetyl-CoA metabolism by maintaining the acetyl-CoA/CoA ratio in the cell and thereby regulating pyruvate dehydrogenase activity (Sawicka et al., 2020). In the present study, acetylcarnitine emerged as one of the most intensity-dependent metabolites, whereas no other acylcarnitines such as butyrylcarnitine were found to be intensity dependent. During intense exercise, the capacity of L-carnitine to regulate acetyl-CoA may be exceeded, leading to acetyl-CoA accumulation and subsequent acetylation of amino acids. However, because of the limited understanding of Ac-AA in the context of sports and exercise, this hypothesis remains speculative and requires further research.

All seven analyzed acylcholines were identified as significantly intensity-dependent, systematically decreasing with exercise intensity. Acetylcholine, the most prominent acylcholine, acts as a neurotransmitter with numerous physiological functions (Picciotto et al., 2012). However, the physiological functions of other acylcholines remain largely unknown. The proposed target enzyme for most acylcholines is the widely expressed butyrylcholinesterase (BChE; EC 3.1.1.8) (Kinchen et al., 2022). An increasing body of evidence connects altered levels of circulating acylcholines with various diseases (Audet-Delage et al., 2018; Zeleznik et al., 2018) and BChE activity with body weight, BMI, and systolic blood pressure (Valle et al., 2011). Recently, a modulating role of acylcholines in acetylcholine metabolism has been hypothesized (Kinchen et al., 2022). The significant and systematic intensity-dependent decrease in all seven analyzed acylcholines in the present study strongly suggests potential physiological functions. However, their specific functions in exercise metabolism remain unclear and require further research.

Our findings align with previous research, showing that metabolites related to energy metabolism, such as TCA cycle intermediates, glycolysis end-products, and acylcarnitines, increase with exercise (Schranner et al., 2020). However, amino acid metabolism emerged as the most intensity-dependent pathway, yet we did not observe significant changes in proteinogenic amino acids. In contrast, a recent cycling study in professional athletes reported increases in proteinogenic amino acids (Nemkov et al., 2023). Differences in study protocols have been suggested to account for varying findings related to proteinogenic amino acids (Schranner et al., 2020); however, our results suggest that exercise intensity is not likely to explain these inconsistencies. Moreover, recent research has shown that β-hydroxybutyrate (BHB), like lactate, can be conjugated with free amino acids via the same enzyme, CNDP2, to form BHB-amino acids, that may exert effects similar to Lac-Phe (Moya-Garzon et al., 2025). In our study, BHB, previously described as a marker of endurance exercise (Morville et al., 2020), did not show statistically significant intensity-dependent changes, possibly due to strict multiple testing correction.

Our results indicate that exercise intensity significantly influences exercise-induced metabolic alterations. Consequently, protocols with dissimilar exercise intensities may not be comparable and future studies should carefully select exercise intensity to ensure that the intended metabolic response can be accurately observed. Exerkines such as Lac-Phe, lactate, and succinate stand out as some of the most intensity-dependent metabolites and may not accumulate substantially in response to moderate-intensity exercise. Therefore, exerkine-mediated physiological effects such as the potential anti-obesity effects of Lac-Phe may only be present following vigorous or high exercise intensities. These findings support the need for intensity-based exercise guidelines and offer implications for targeted exercise recommendations. Moreover, exerkines may be closely associated with higher exercise intensities, reflecting the increased need for physiological adaptations at these intensities. Consequently, study protocols utilizing higher exercise intensities may better facilitate the identification of metabolic candidate exerkines or metabolites with potential physiological significance, such as N-acetylated amino acids and acylcholines. However, research on these metabolites is limited, and the underlying mechanisms driving their systematically altered levels in response to vigorous-intensity exercise as well as their origins remain unclear and require further research.

To the best of our knowledge, this is the first untargeted metabolomics study to investigate and compare metabolic changes in blood samples in response to different exercise intensities using a crossover design. A crossover study design was employed to minimize bias due to inter-individual variability and multiple plasma samples were collected to obtain a detailed picture of post-exercise metabolic alterations. The metabolomics analyses used ensured a highly standardized analytical approach and led to the identification of over 1000 metabolites, which provided an extensive picture of the metabolome. However, the absolute concentrations of metabolites could not be determined in an untargeted metabolomics approach. Data preparation and analysis were performed using established statistical methods and robust linear mixed models. Participants adhered to standardized isocaloric diets but the possibility that the pre-exercise breakfast altered the participants’ metabolome, particularly due to variations in protein or carbohydrate intake, cannot be discounted. The homogeneous study population of young, healthy, and physically active men enhanced comparability among participants but limited the generalizability of the results to the broader population and precluded conclusions about gender differences.

## Conclusion

This study aimed to investigate the effects of exercise intensity on the metabolome of young, healthy, and physically active men following 30 min of moderate-intensity and vigorous-intensity aerobic exercise. Numerous metabolites primarily involved in amino acid metabolism and energy conversion pathways, including N-lactoyl-amino acids, TCA cycle intermediates, N-acetylated amino acids, and acylcholines, were identified as intensity dependent. Our findings indicate that exercise intensity plays a crucial role in shaping the metabolic response to physical activity. Exerkines such as Lac-Phe, lactate, and succinate were among the most intensity-dependent metabolites, and may not be synthesized in substantial amounts following moderate-intensity exercise. Moreover, Ac-AA and acylcholines were systematically altered by exercise intensity, indicating potential physiological functions. These results strongly link exerkine responses to exercise intensity, offering valuable insights for targeted exercise recommendations and physical activity guidelines.

## Supplementary Information

Below is the link to the electronic supplementary material.

Supplementary file1 (DOCX 3499 KB)

Supplementary file2 (DOCX 966 KB)

Supplementary file3 (DOCX 658 KB)

Supplementary file4 (DOCX 15 KB)

Supplementary file5 (DOCX 665 KB)

Supplementary file6 (XLSX 38 KB)

Supplementary file7 (XLSX 21 KB)

## Data Availability

All data will be made available upon reasonable request to the corresponding author.
