# Supplementary material for: Exercise intensity determines circulating levels of Lac-Phe and other exerkines: a randomized crossover trial
Source: Metabolomics. 2025 May 7;21(3):63. doi: 10.1007/s11306-025-02260-0 (PMC12058925; doi:10.1007/s11306-025-02260-0)
Supplement: Supplementary file 1 — Supplementary file1 (DOCX 3499 KB) [file 11306_2025_2260_MOESM1_ESM.docx]

**Exercise intensity determines circulating levels**

**of Lac-Phe and other exerkines:**

**a randomized crossover trial**

Dirk Weber^1^, Paola G. Ferrario^2^, Achim Bub^1,2^

^1^ Institute of Sports and Sports Science, Karlsruhe Institute of Technology, Karlsruhe, Germany,

^2^ Department of Physiology and Biochemistry of Nutrition, Max Rubner-Institute, Karlsruhe, Germany

*Metabolomics (Springer)*

**Corresponding author:**

Dirk Weber

Karlsruhe Institute of Technology (KIT)

Engler-Bunte-Ring 15

76131 Karlsruhe (Germany)

[dirk.weber@kit.edu](mailto:dirk.weber@kit.edu)

# QQ Plot for 3-hydroxylaurate


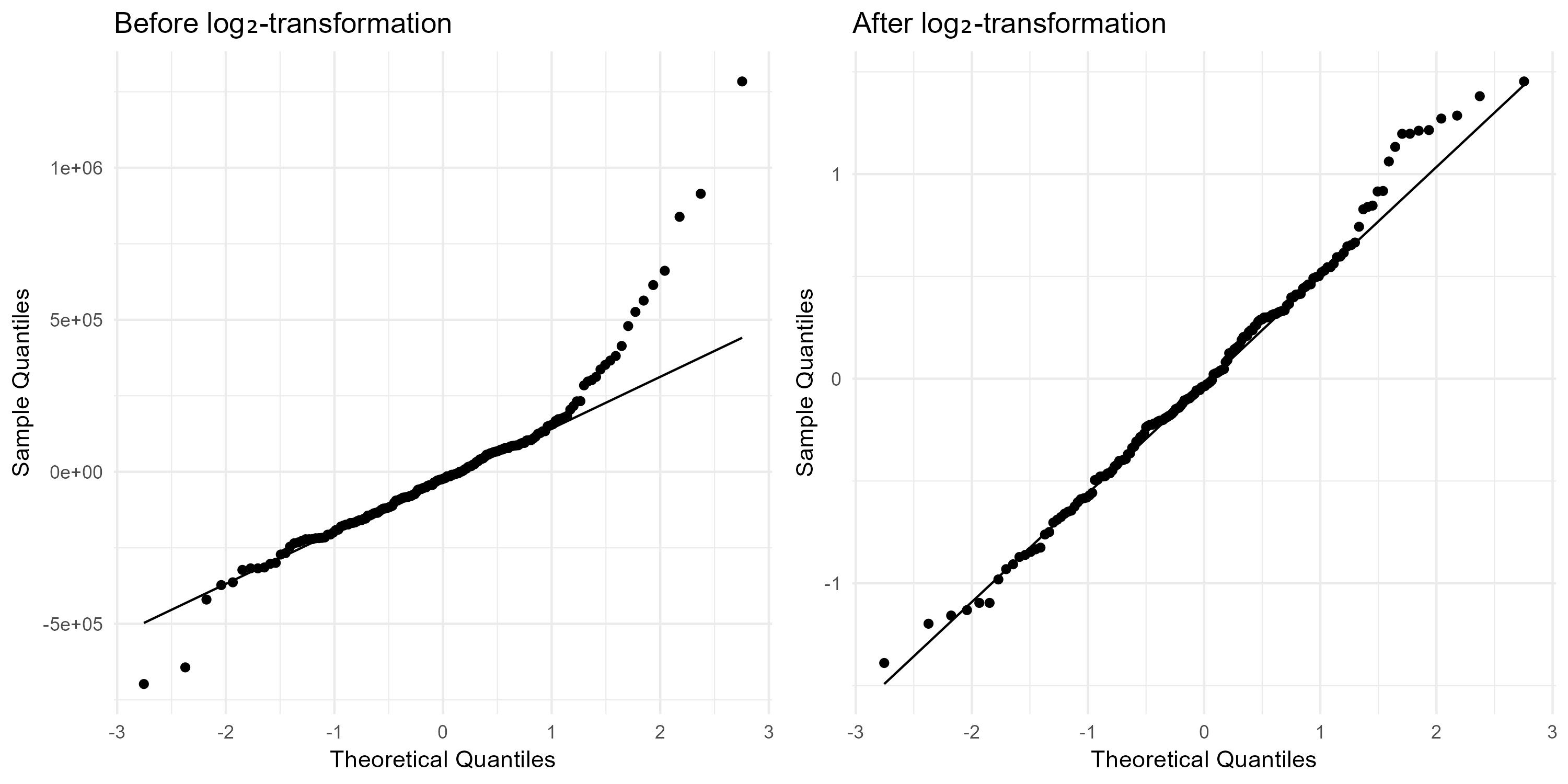


# QQ Plot for 3-(3-amino-3-carboxypropyl)uridine


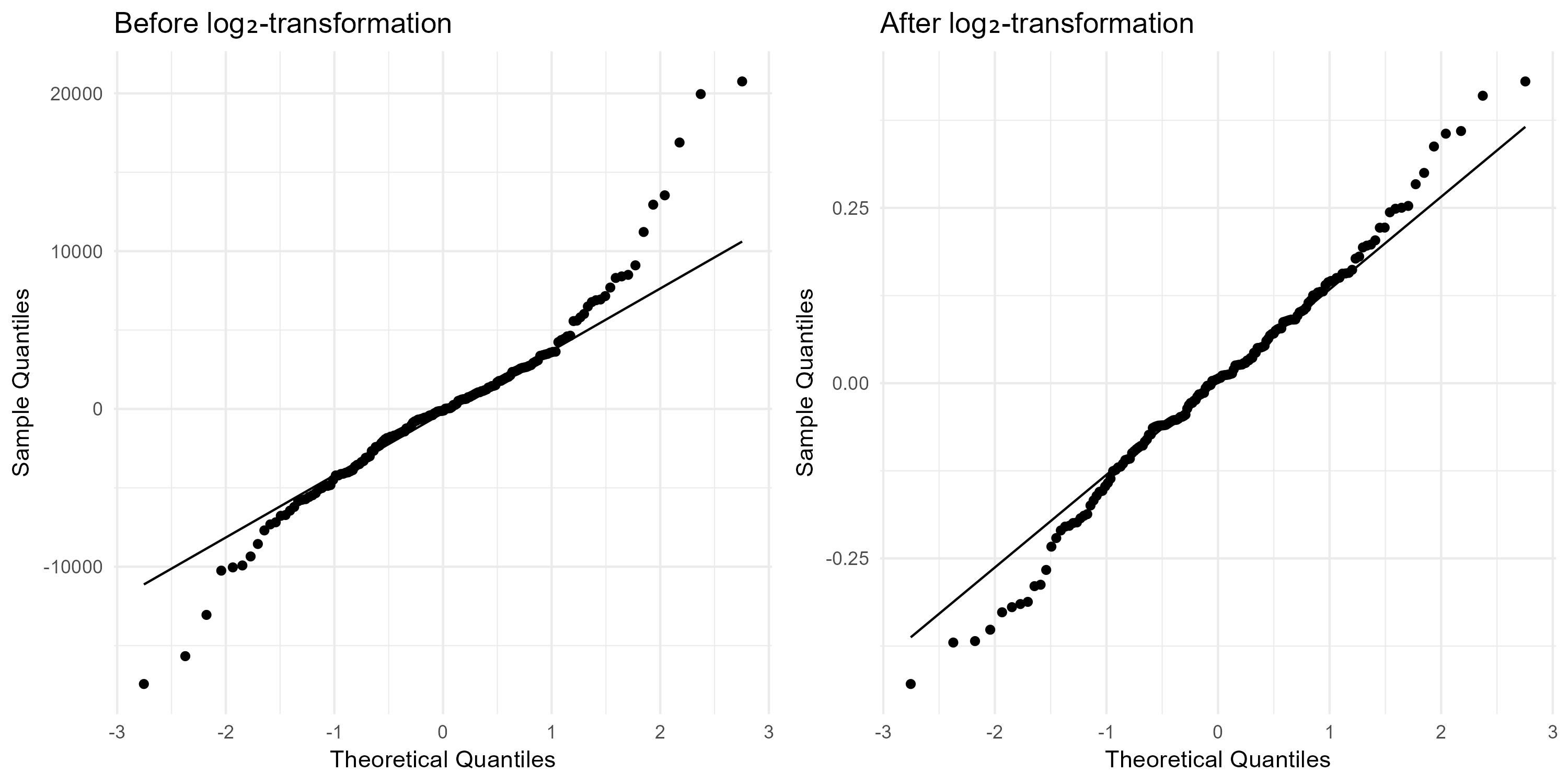


# QQ Plot for 5alpha-androstan-3alpha,17alpha-diol monosulfate


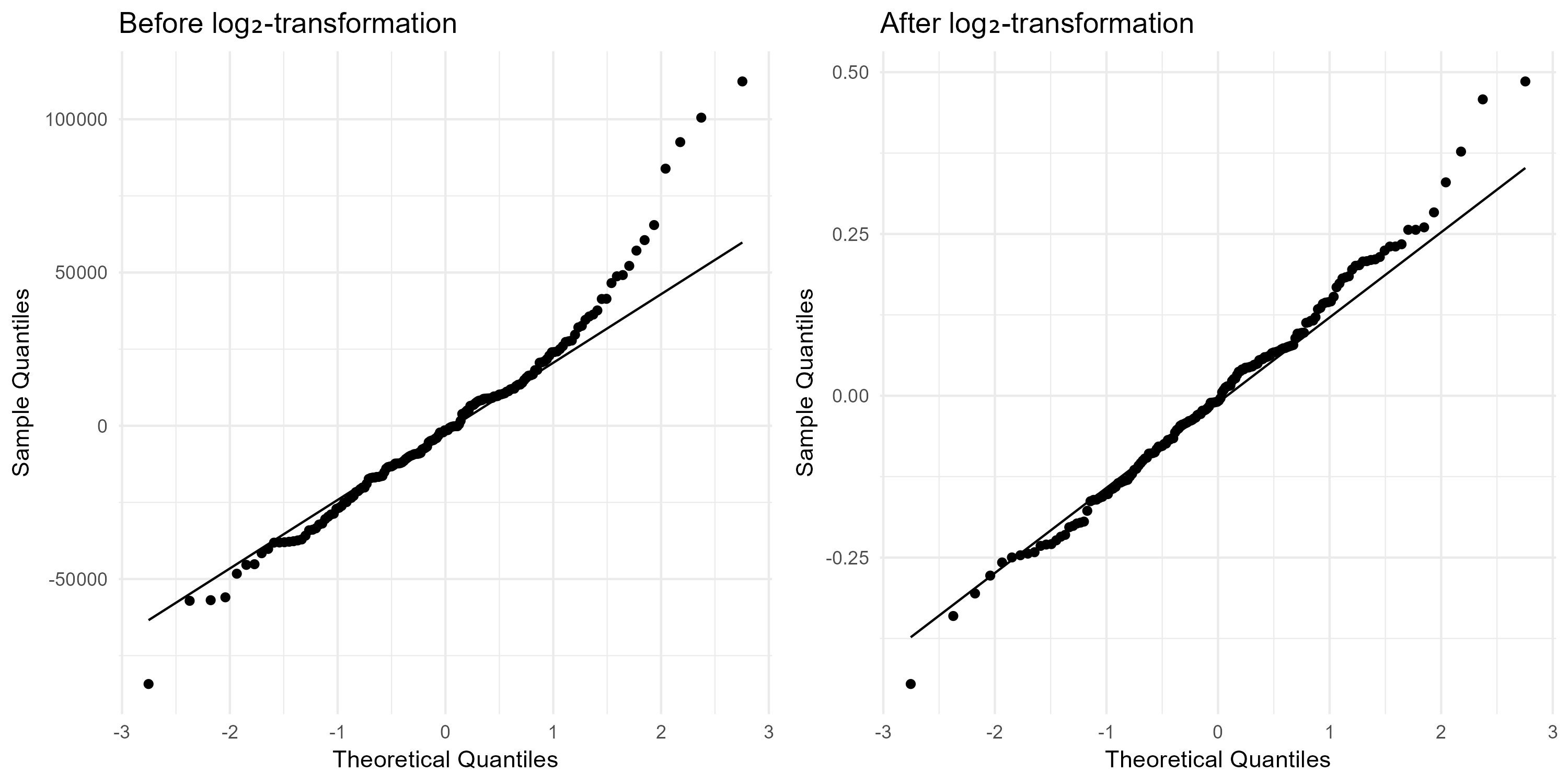


# QQ Plot for erythritol


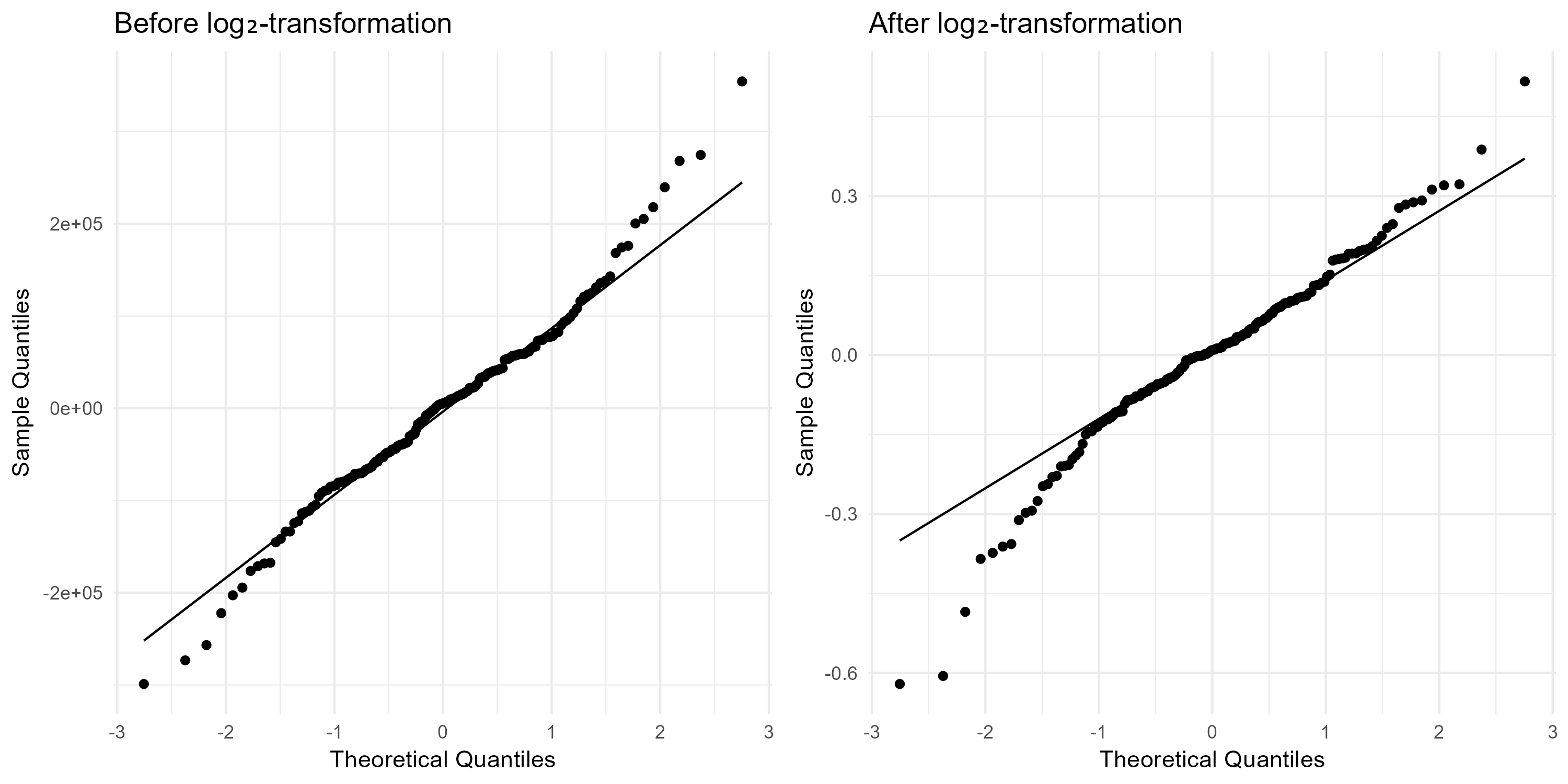


# QQ Plot for 1-oleoyl-2-arachidonoyl-GPI (18:1/20:4)


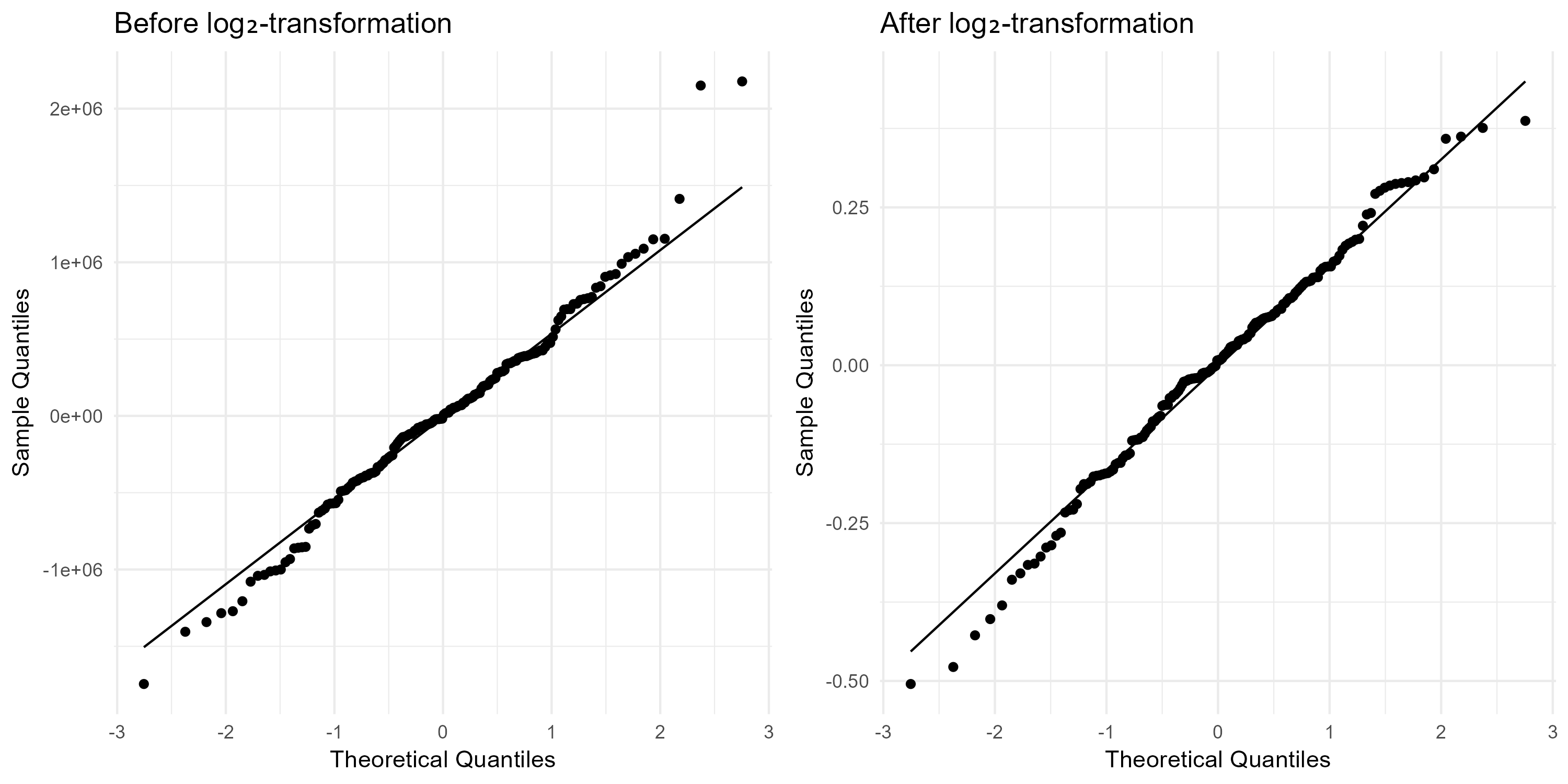


# QQ Plot for myristoleate (14:1n5)


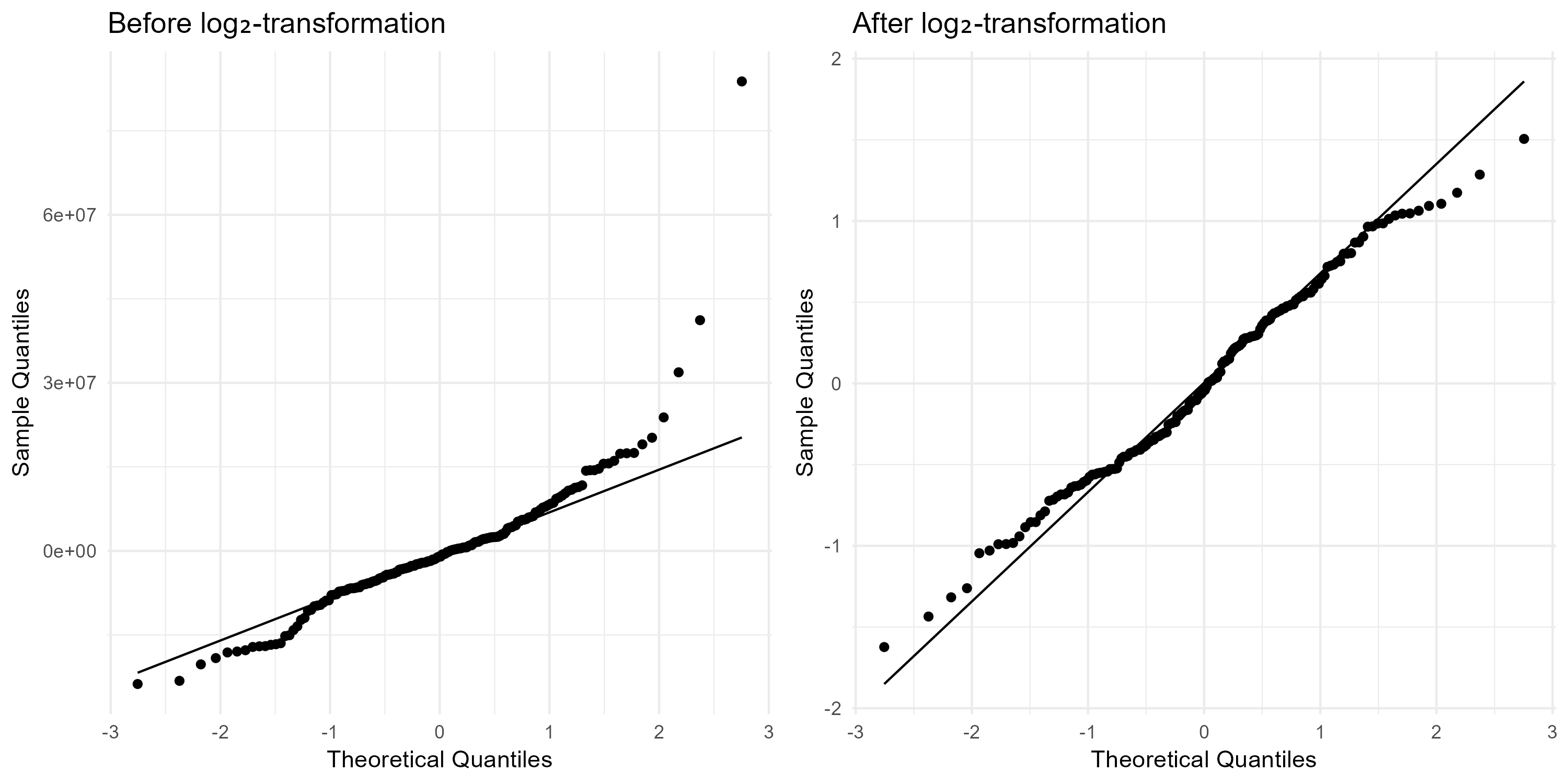


# QQ Plot for X-18921


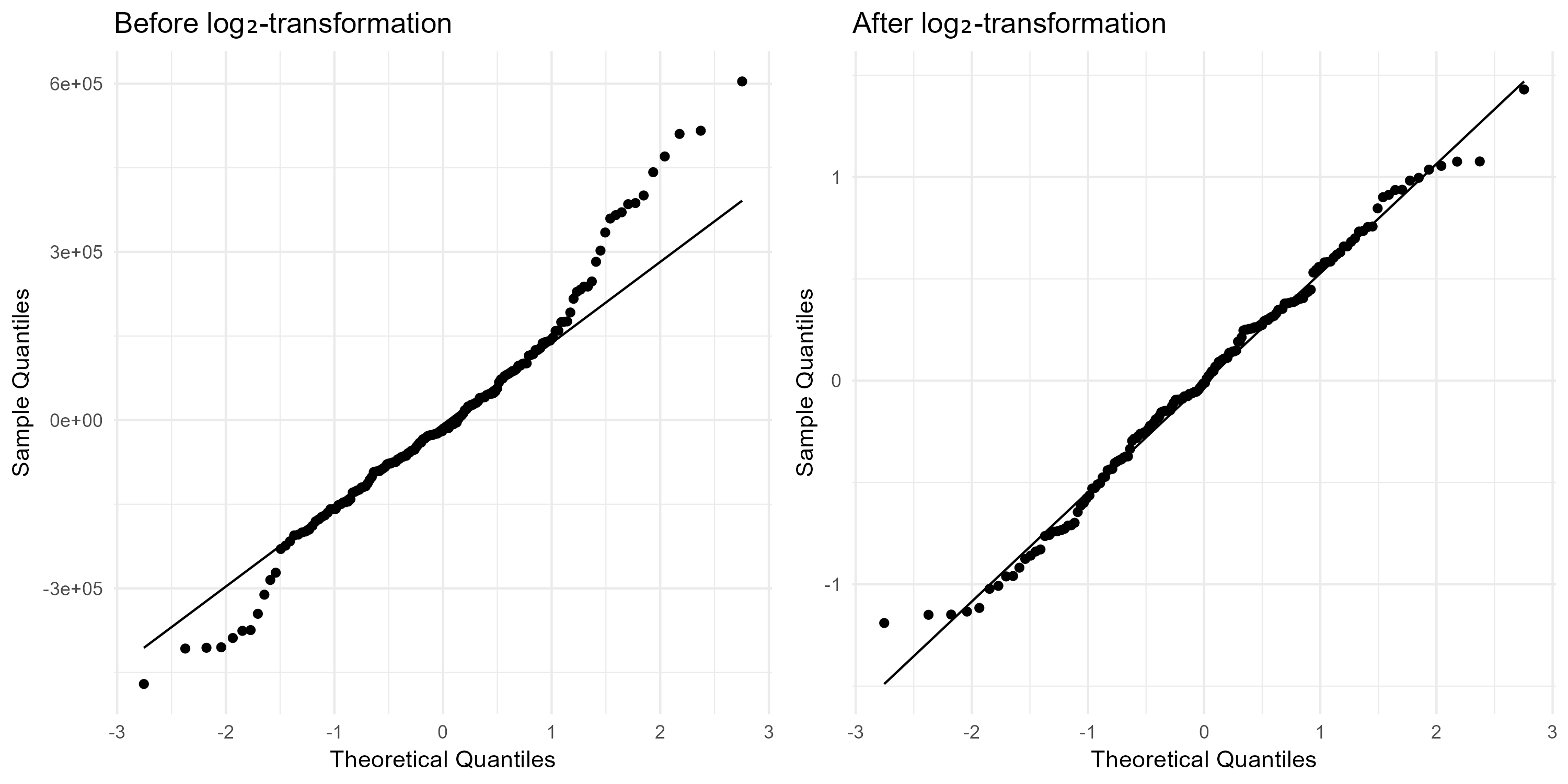


# QQ Plot for X-13866


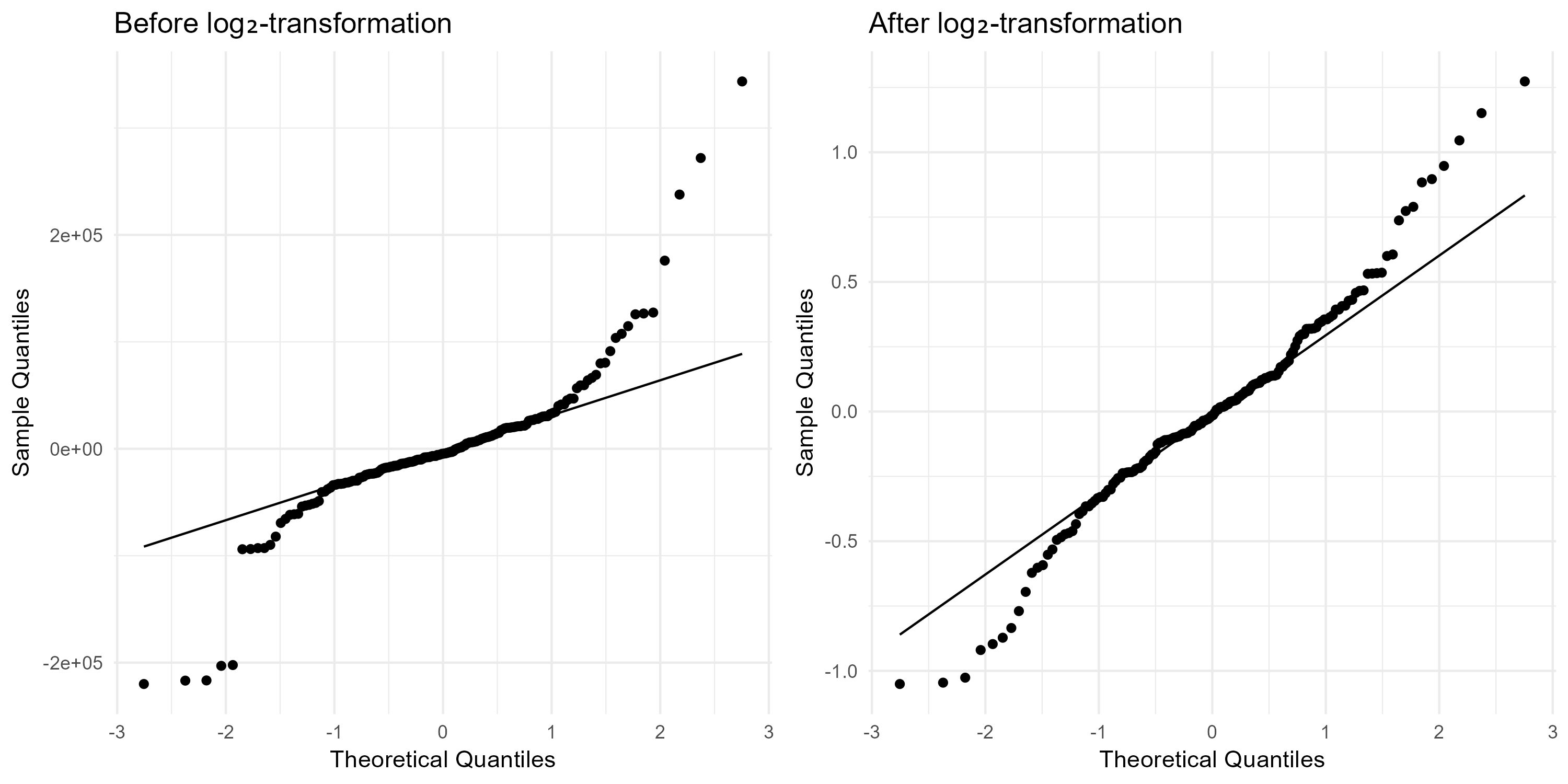


# QQ Plot for citrulline


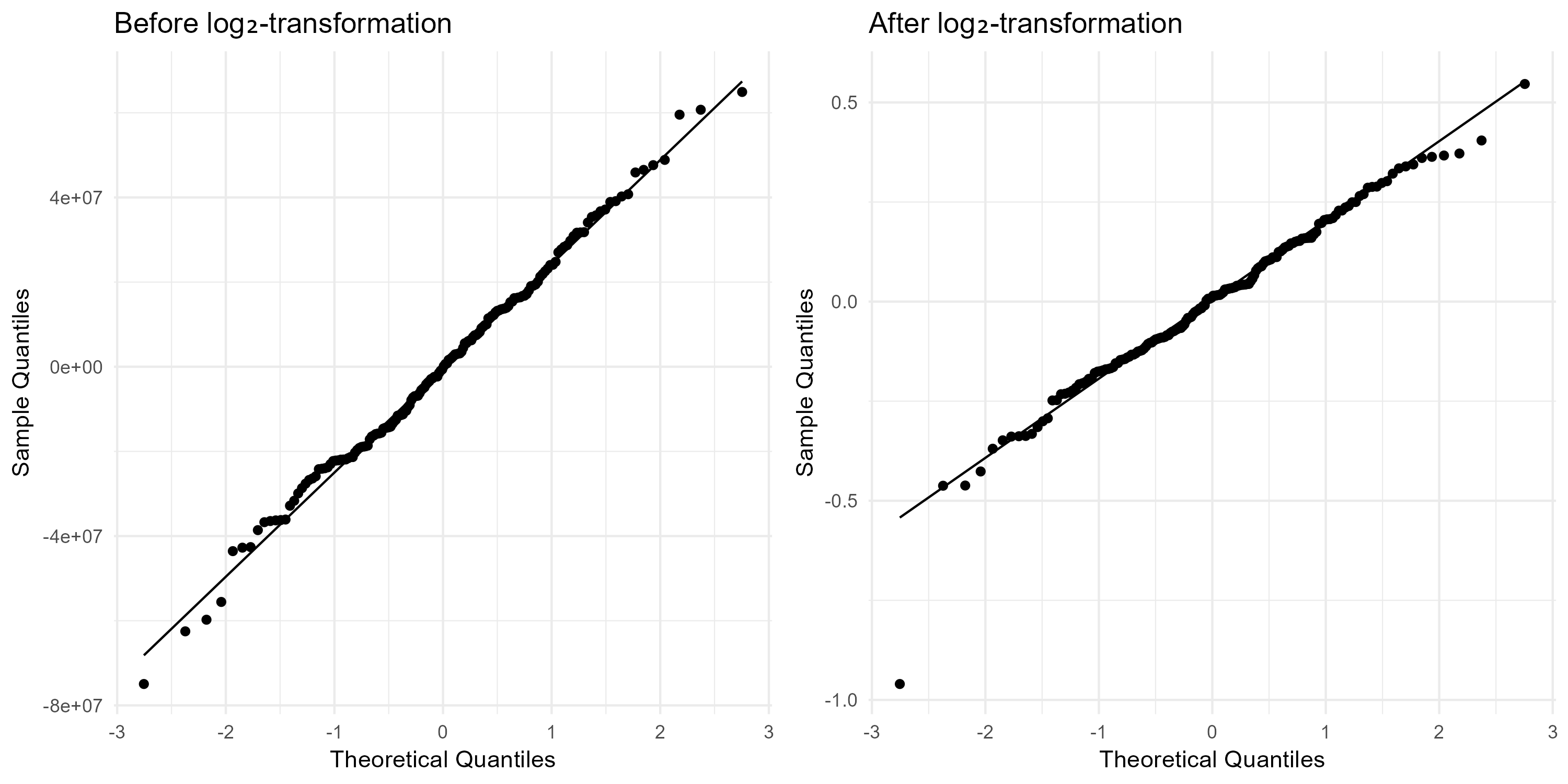


# QQ Plot for lactosyl-N-palmitoyl-sphingosine (d18:1/16:0)


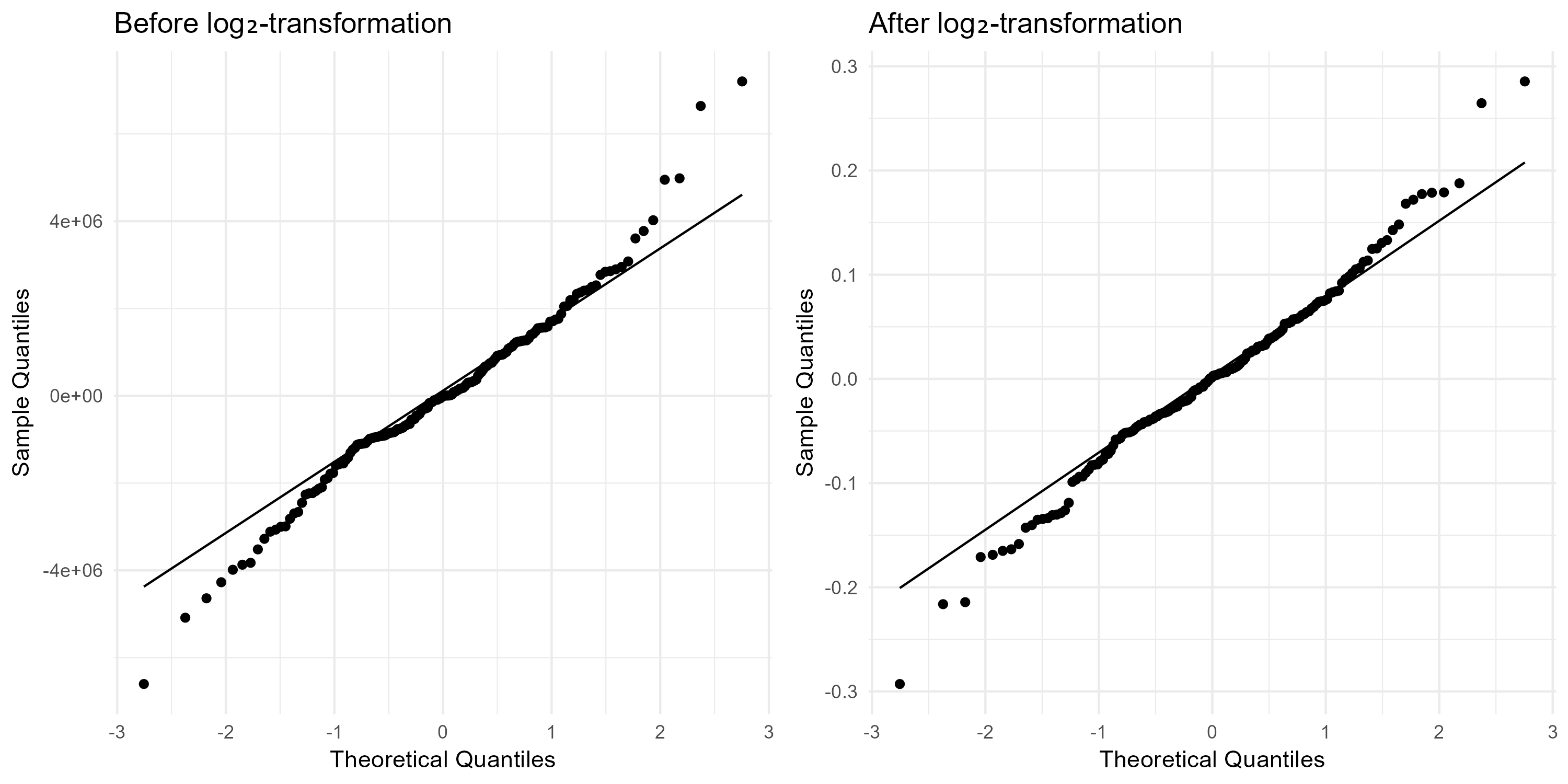


# QQ Plot for galactosylglycerol


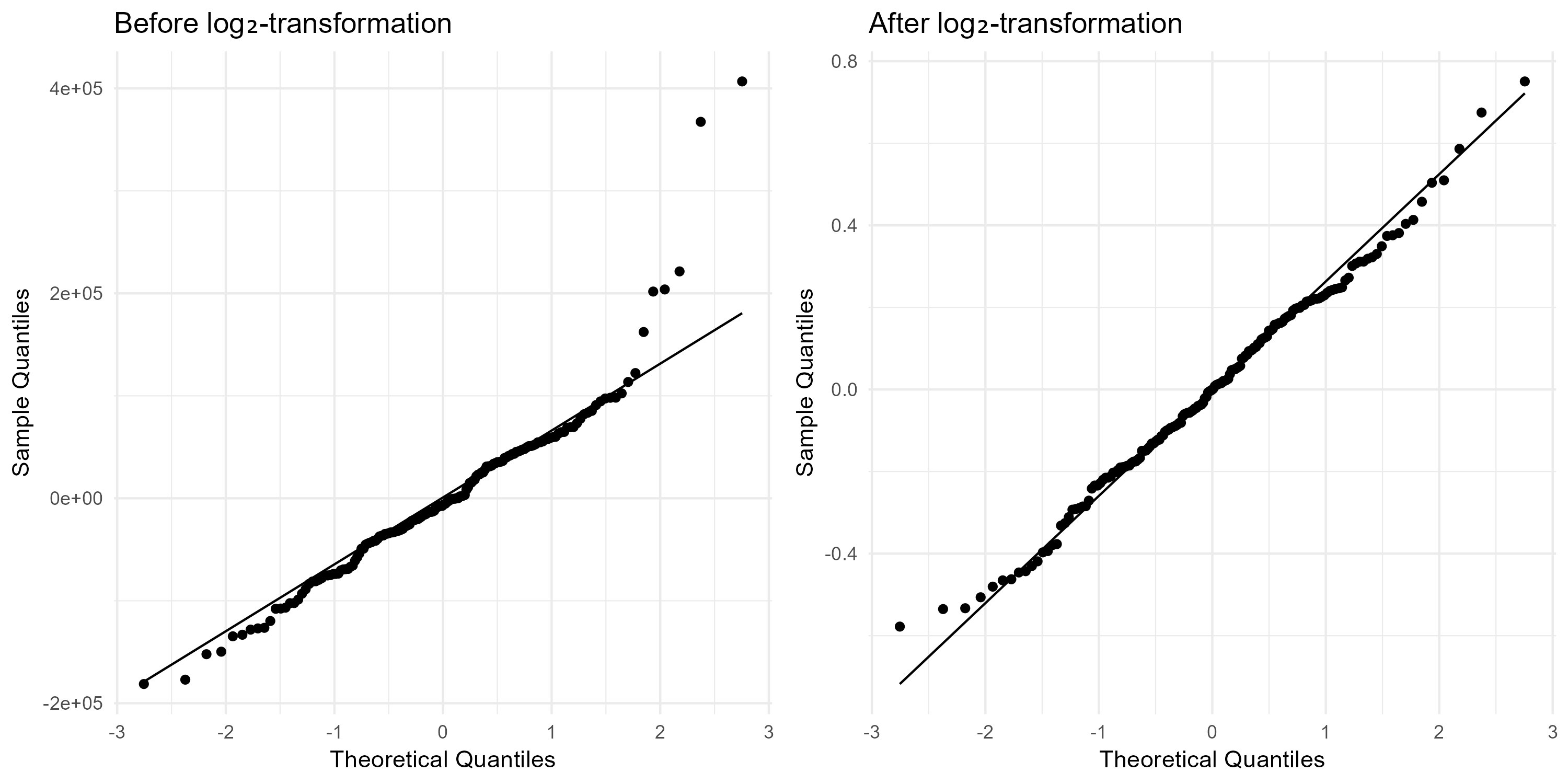


# QQ Plot for sphingomyelin (d18:1/25:0, d19:0/24:1, d20:1/23:0, d19:1/24:0)


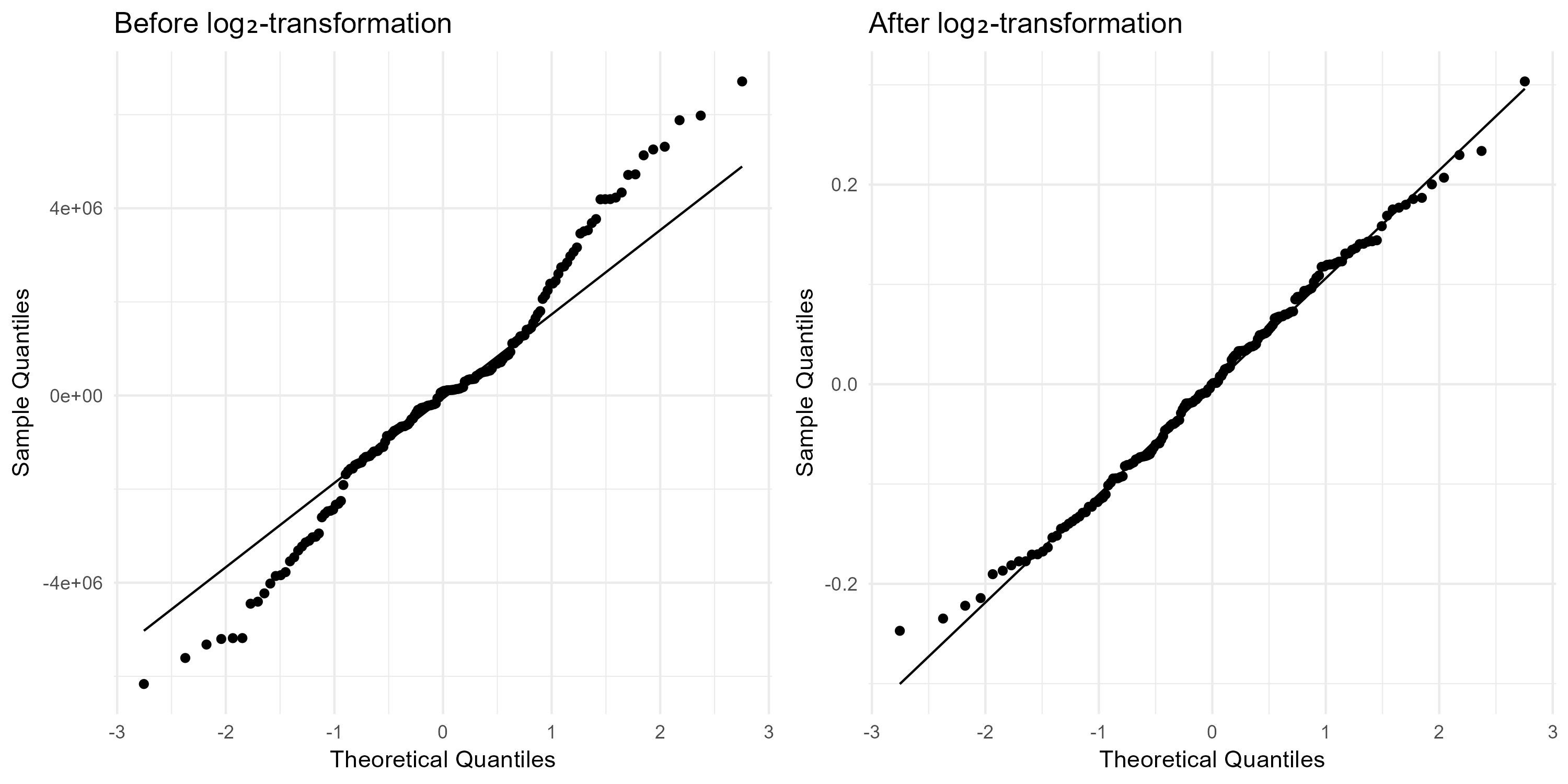


# QQ Plot for 3-methyl-2-oxovalerate


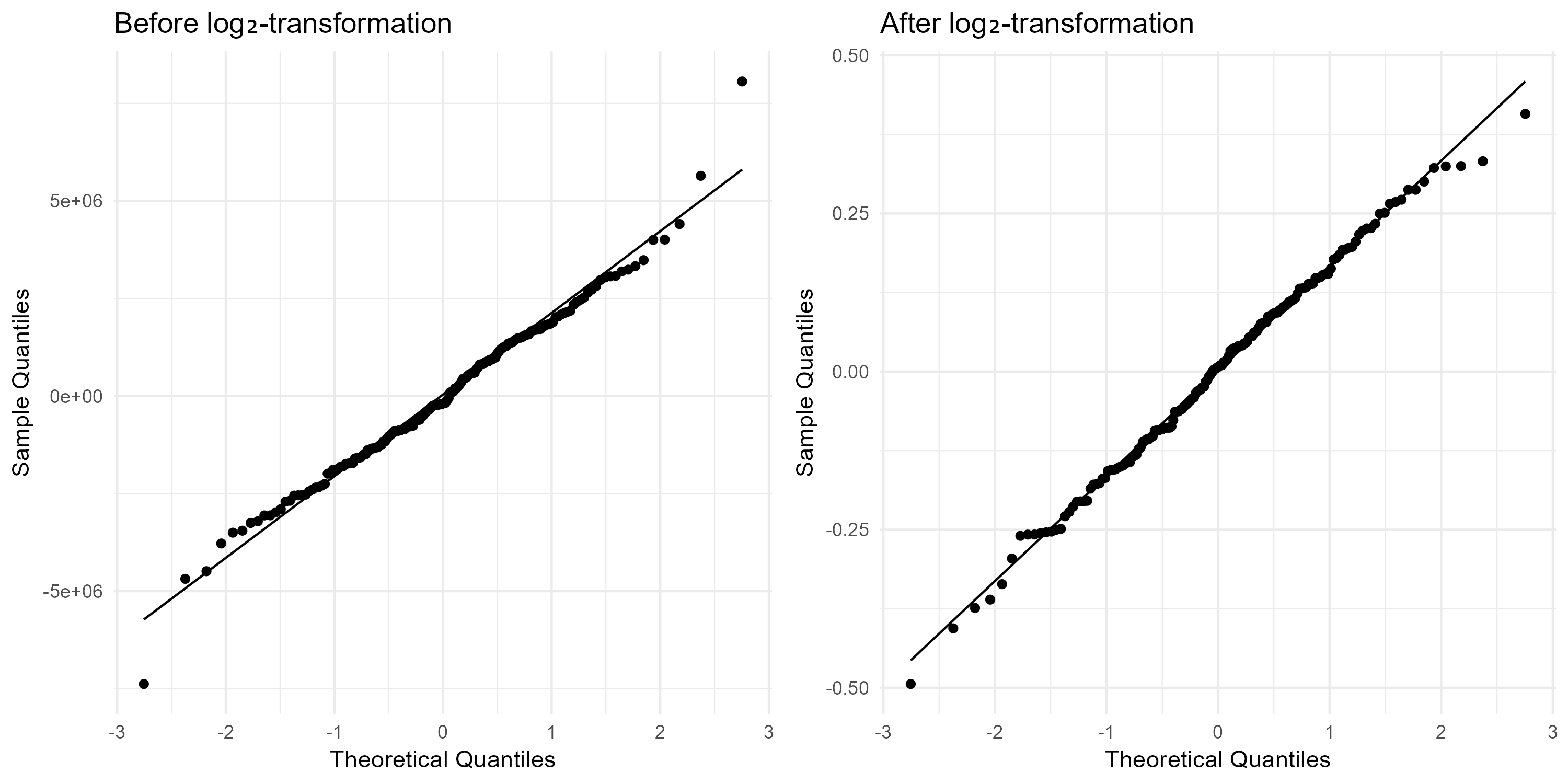


# QQ Plot for X-24947


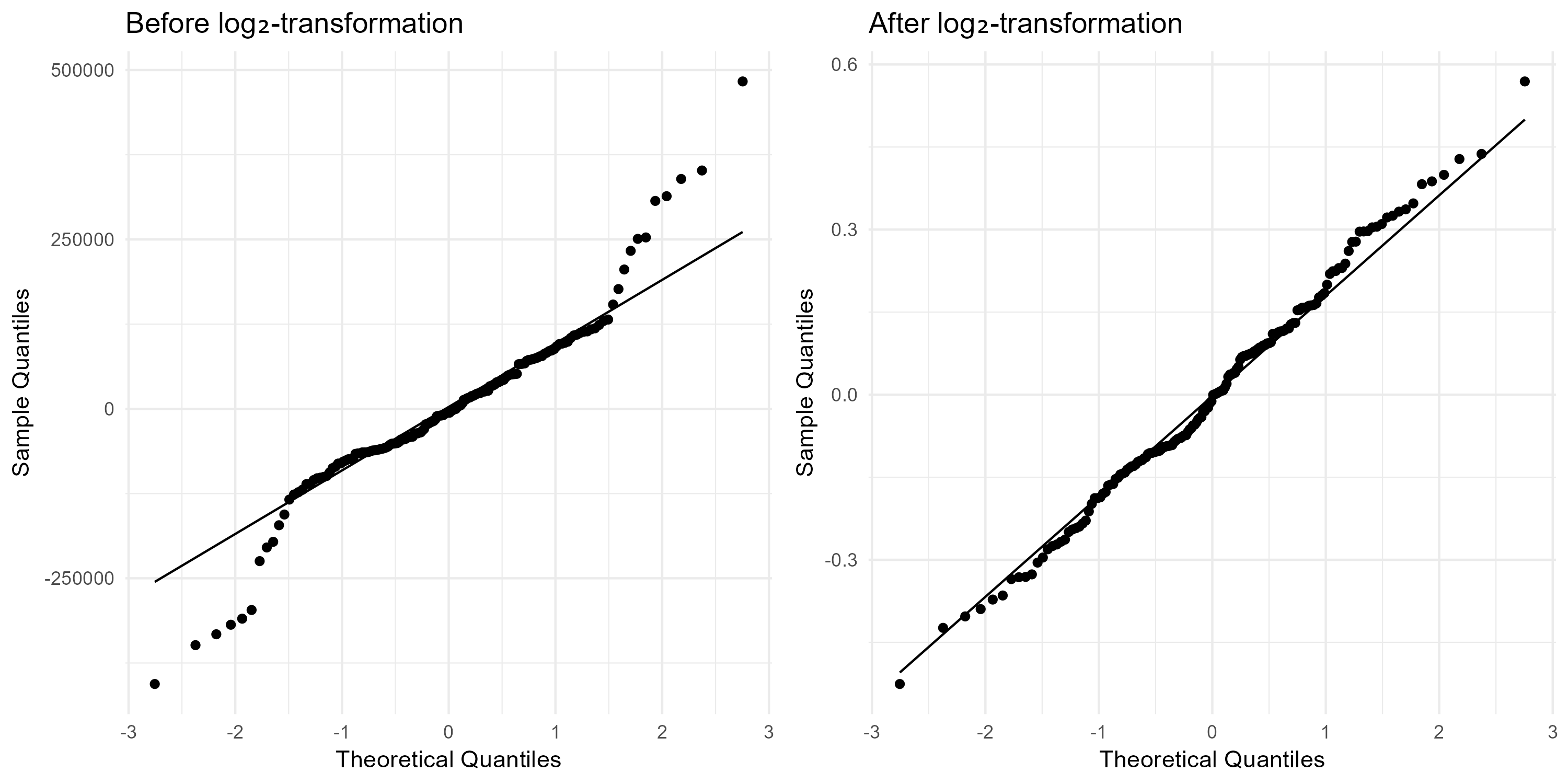


# QQ Plot for alliin


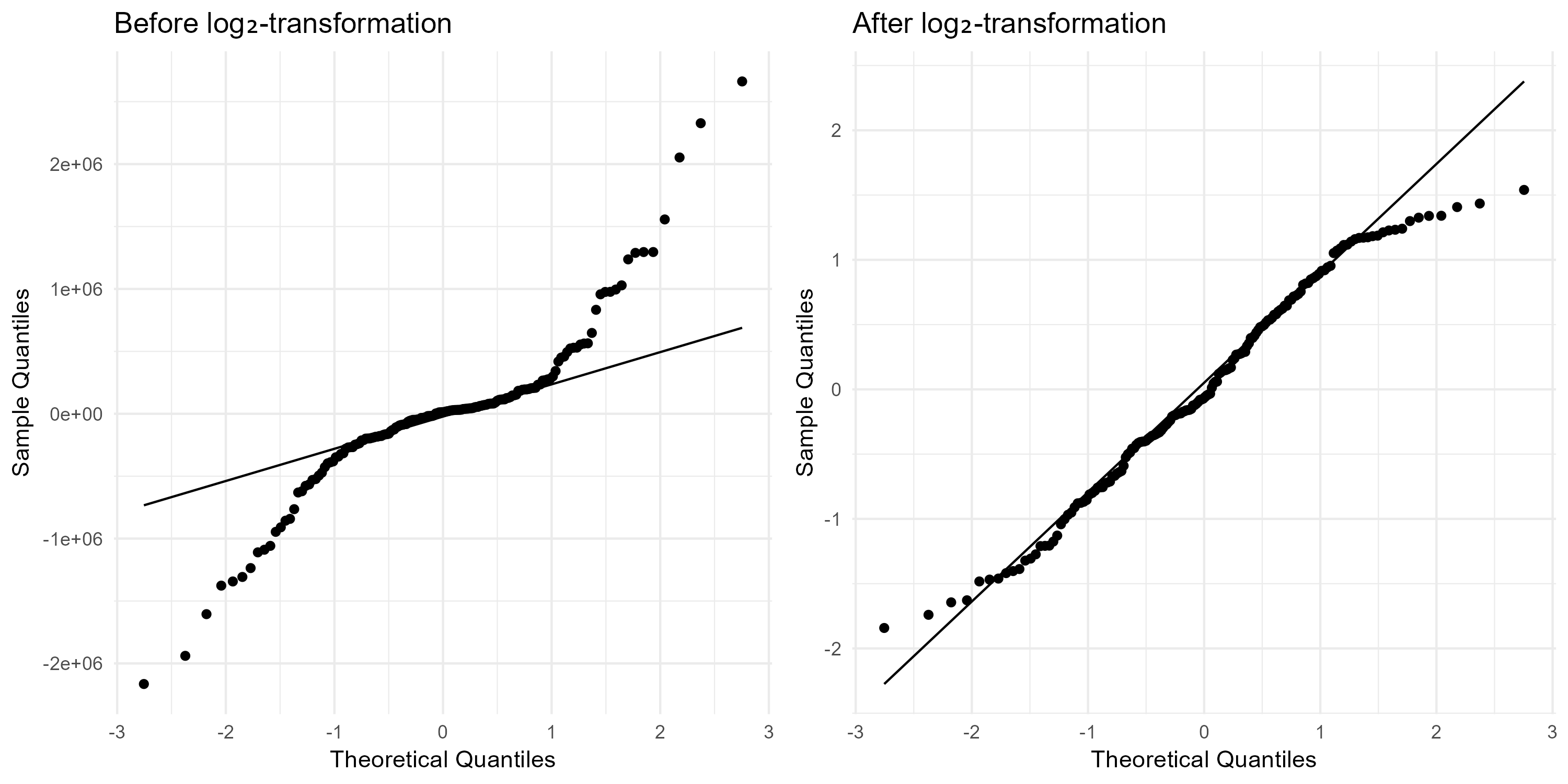


# QQ Plot for X-21283


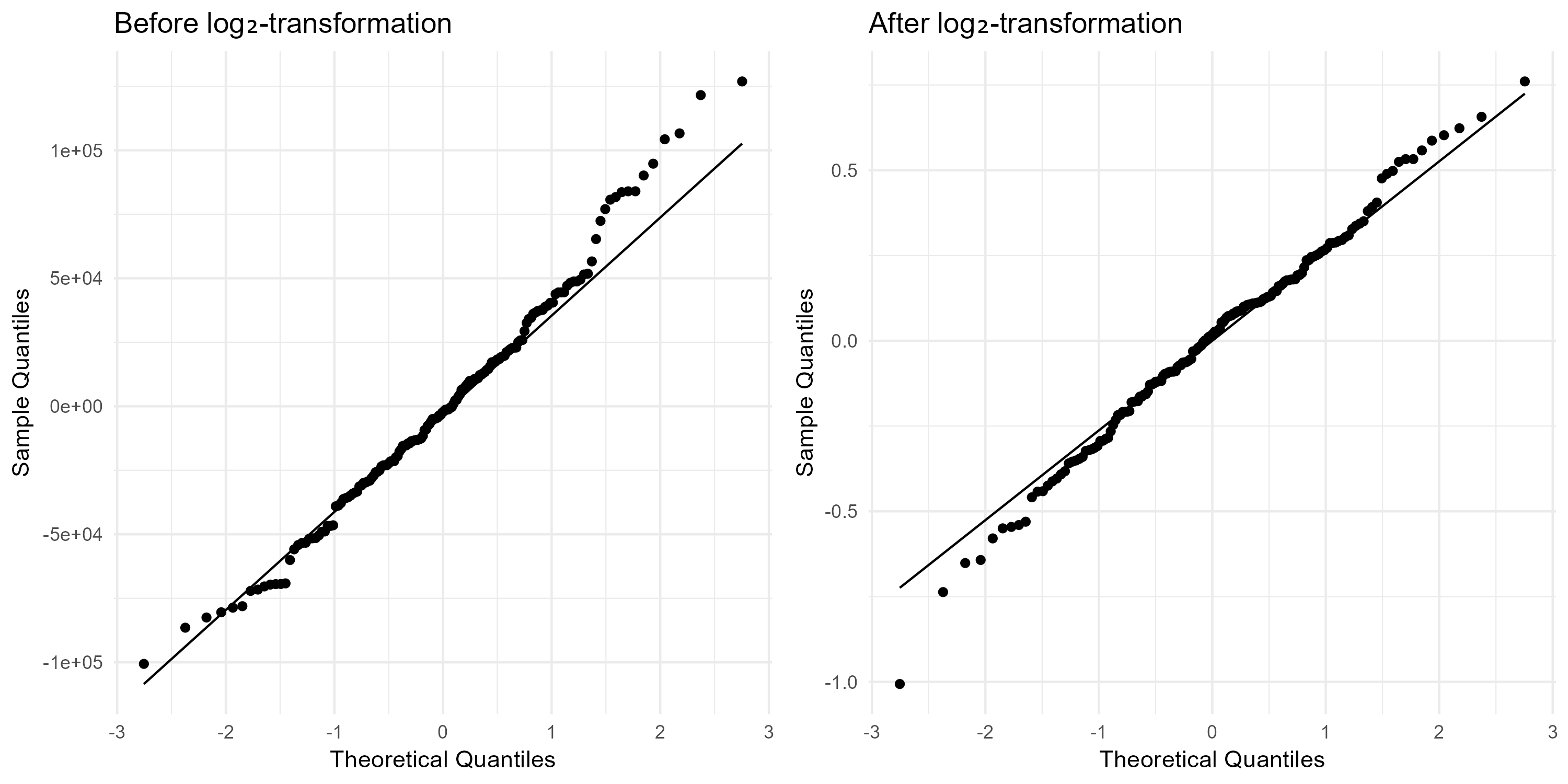


# QQ Plot for X-17010


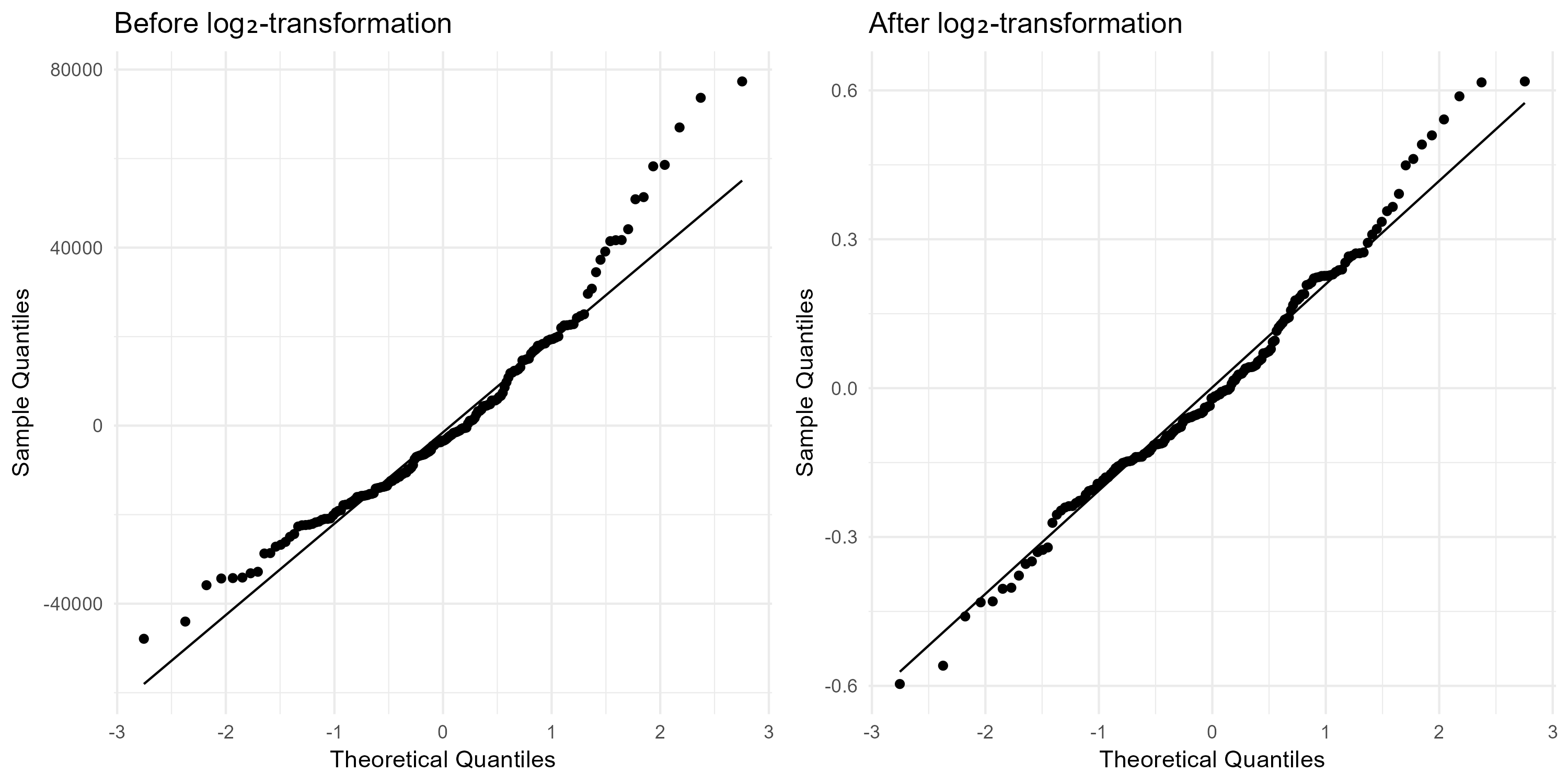


# QQ Plot for glycochenodeoxycholate


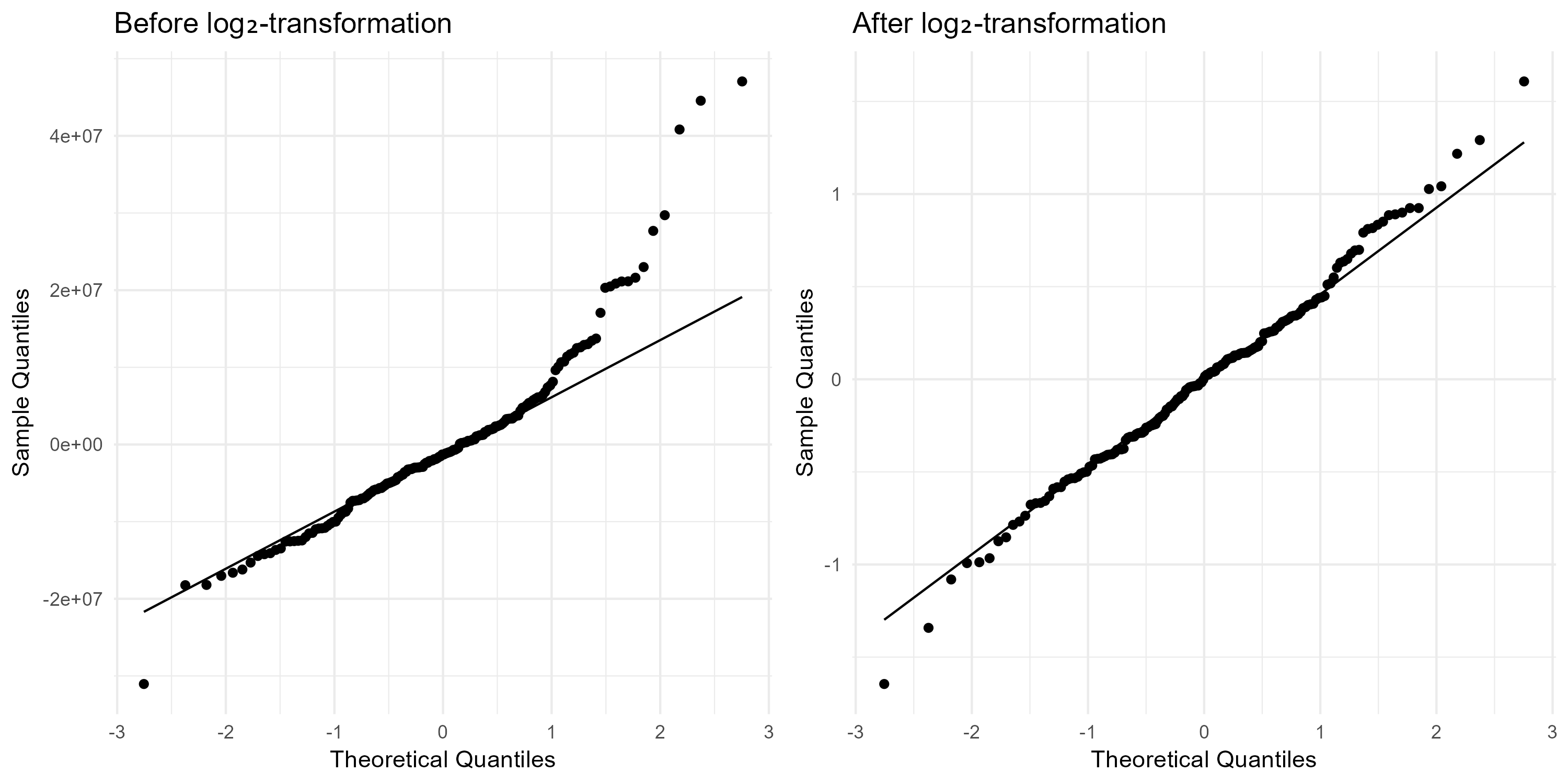


# QQ Plot for 1-(1-enyl-palmitoyl)-2-docosahexaenoyl-GPE (P-16:0/22:6)


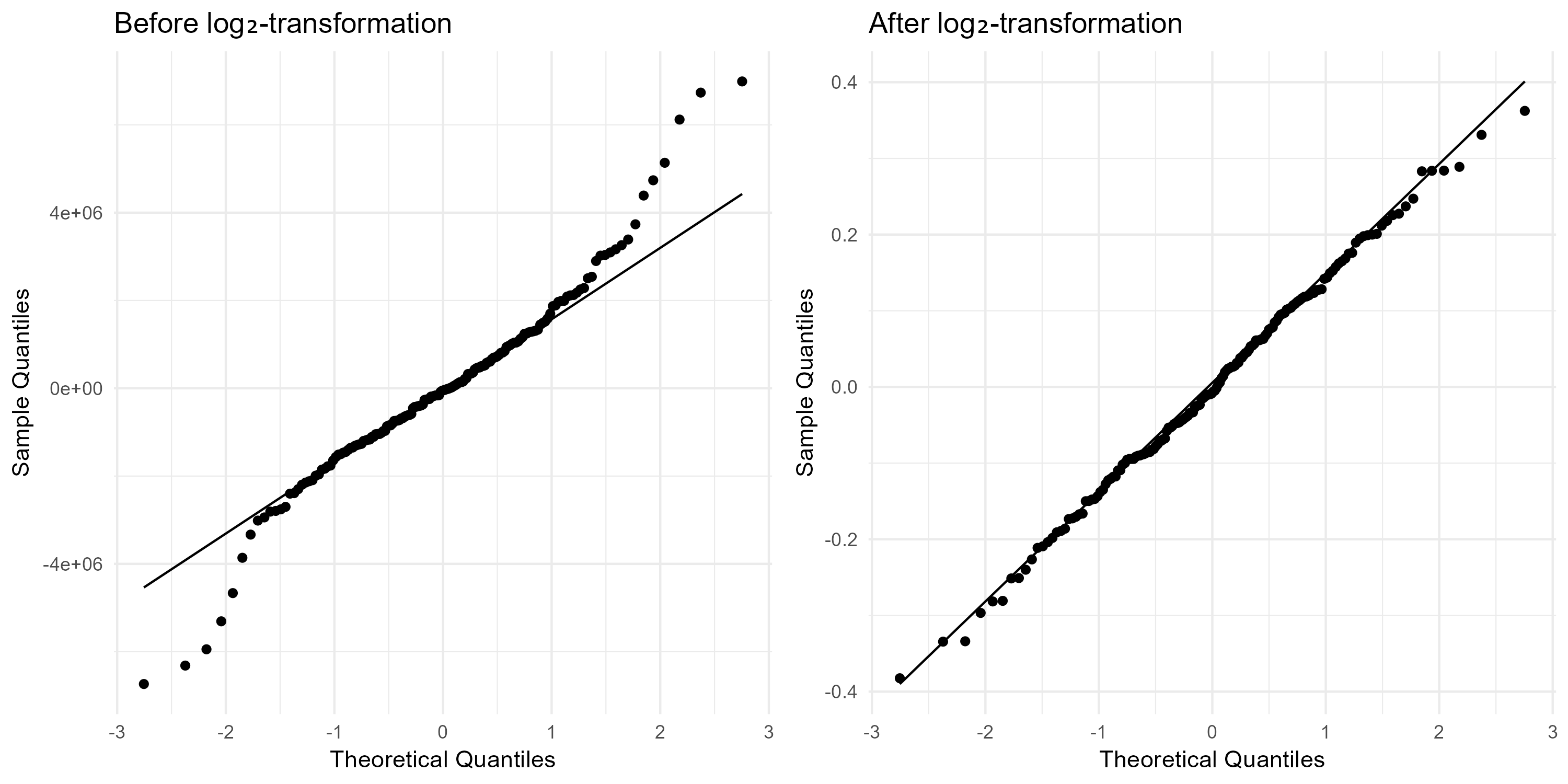


# QQ Plot for dopamine 4-sulfate


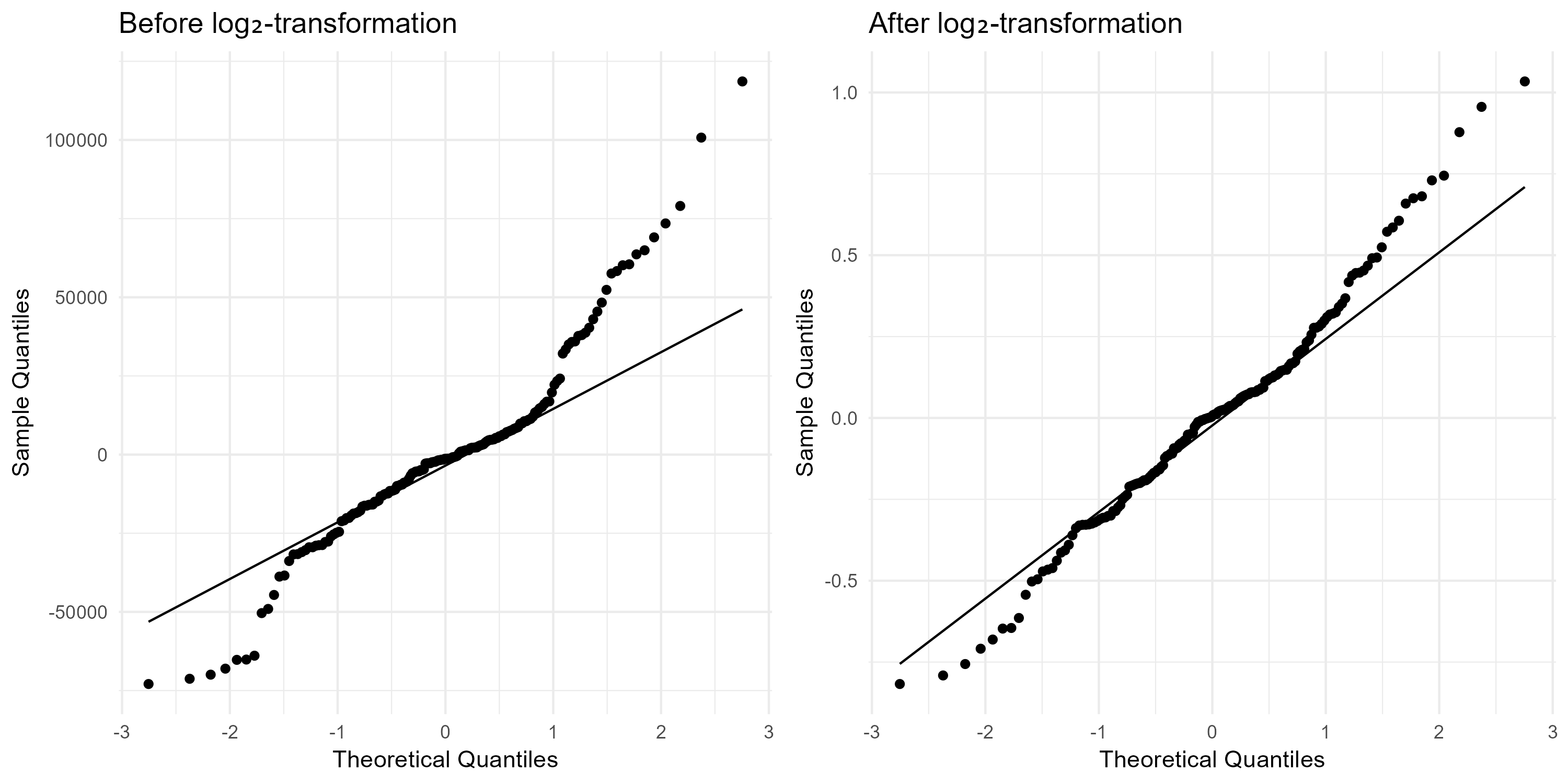


# QQ Plot for nicotinamide


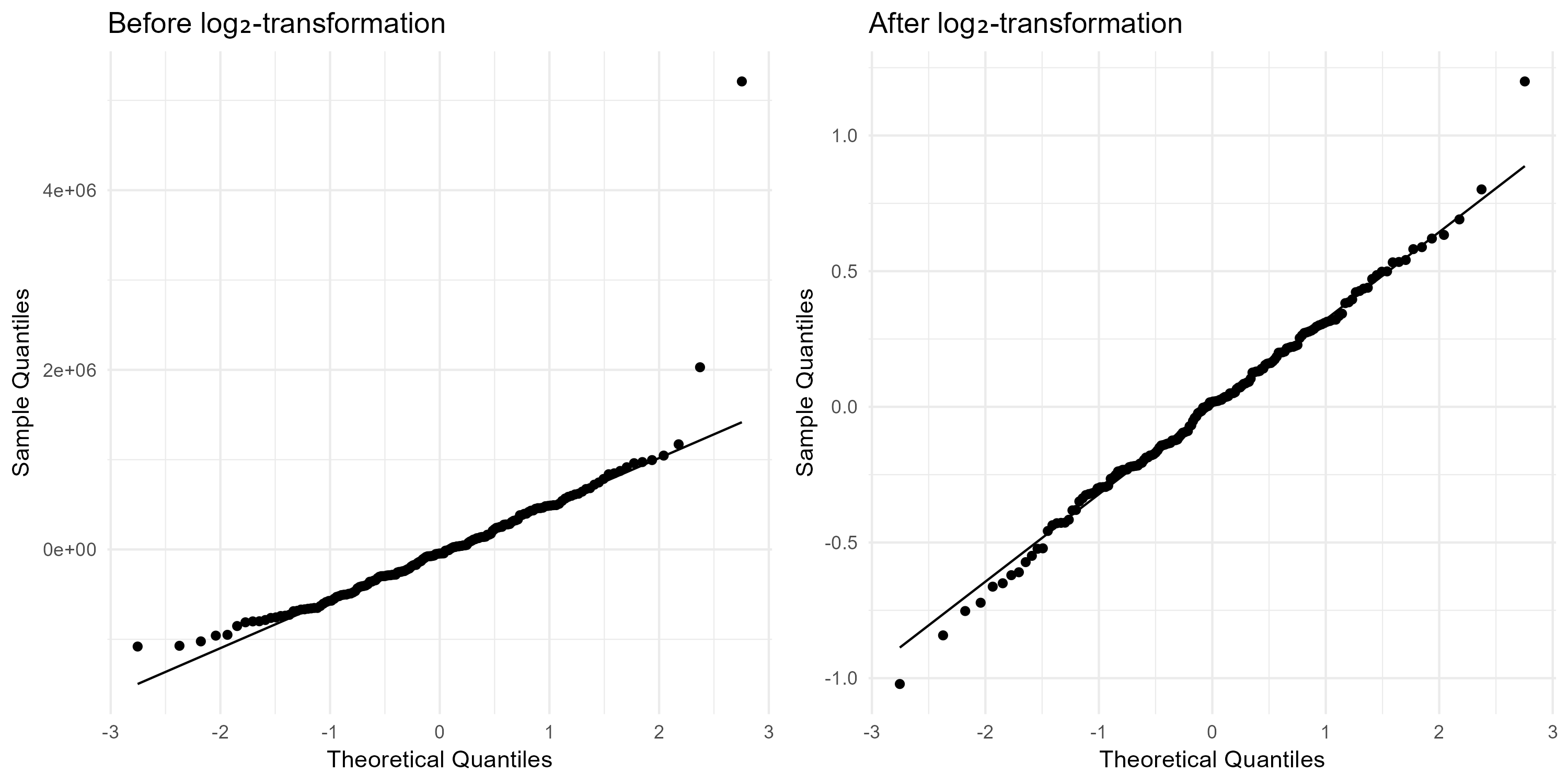


# QQ Plot for 2-keto-3-deoxy-gluconate


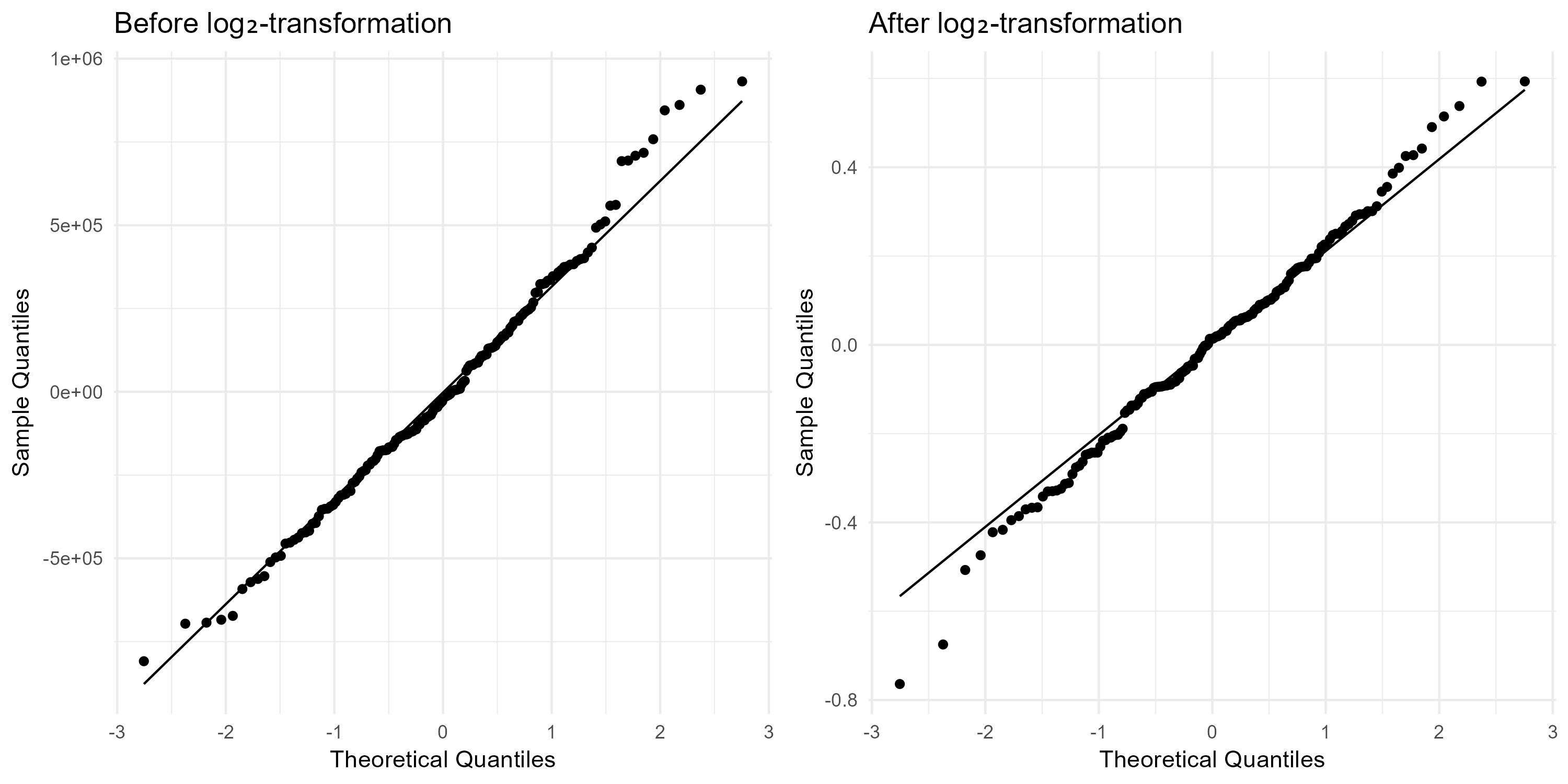


# QQ Plot for 9,10-DiHOME


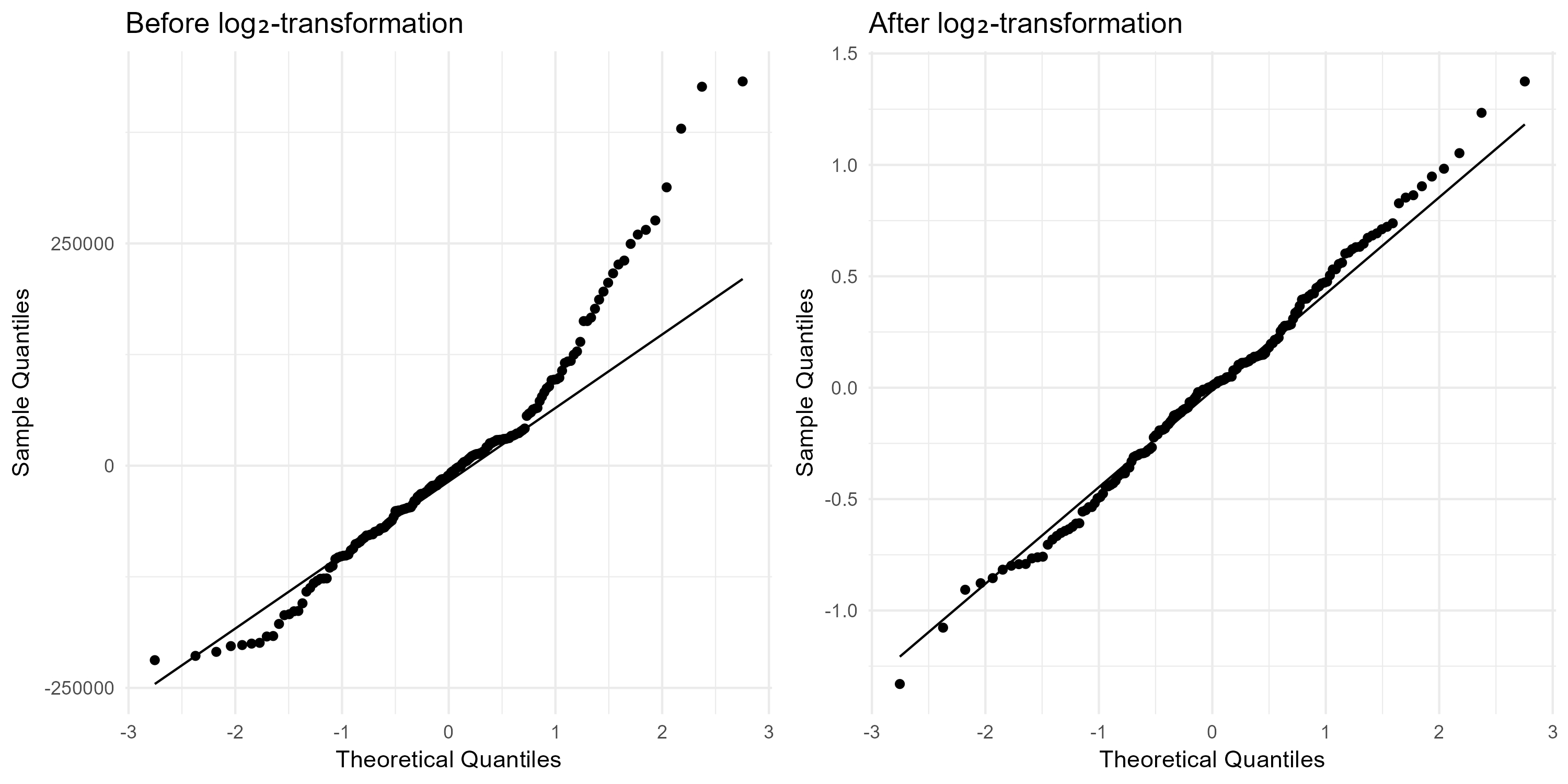


# QQ Plot for taurolithocholate 3-sulfate


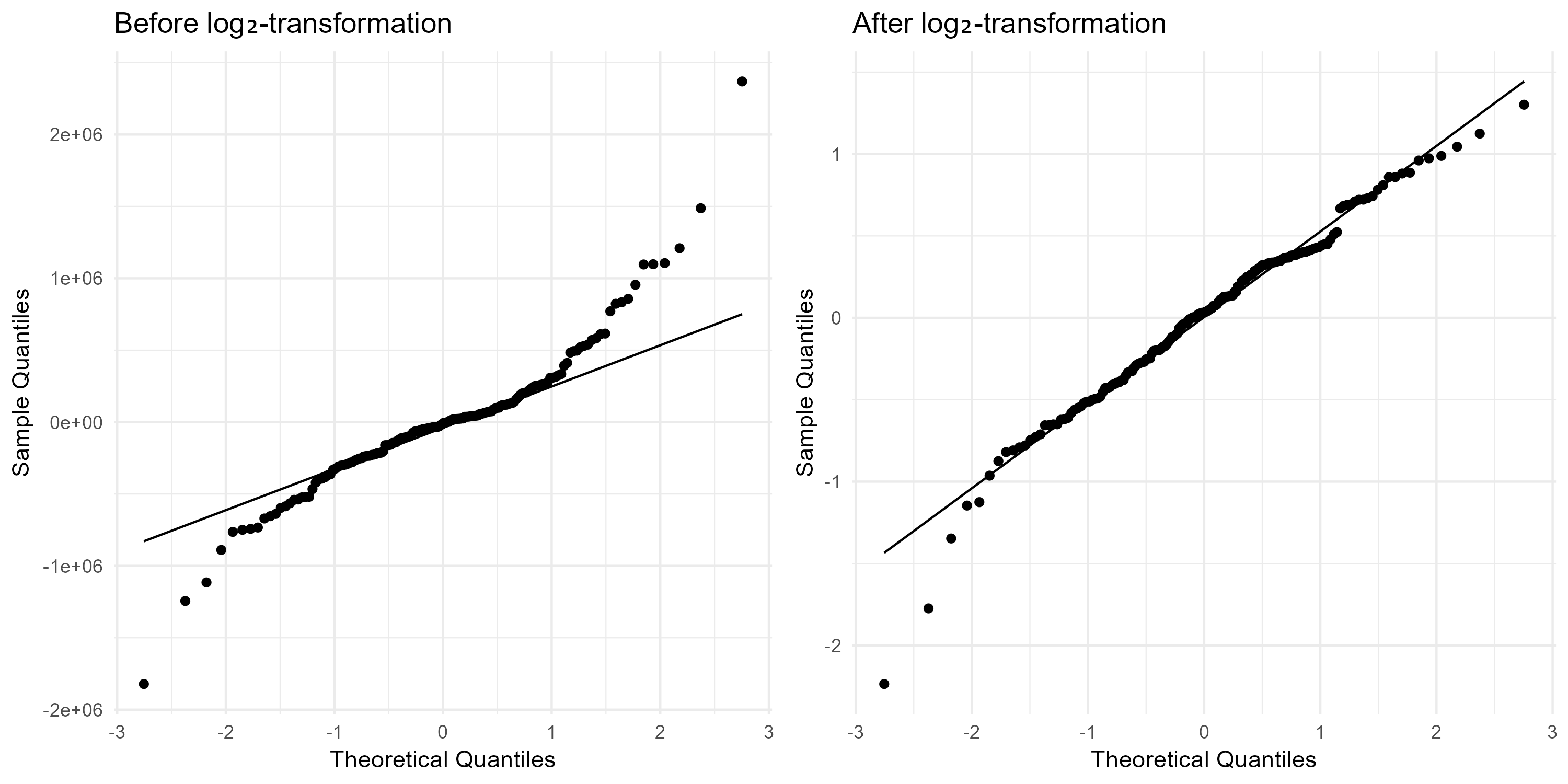


# QQ Plot for pyruvate


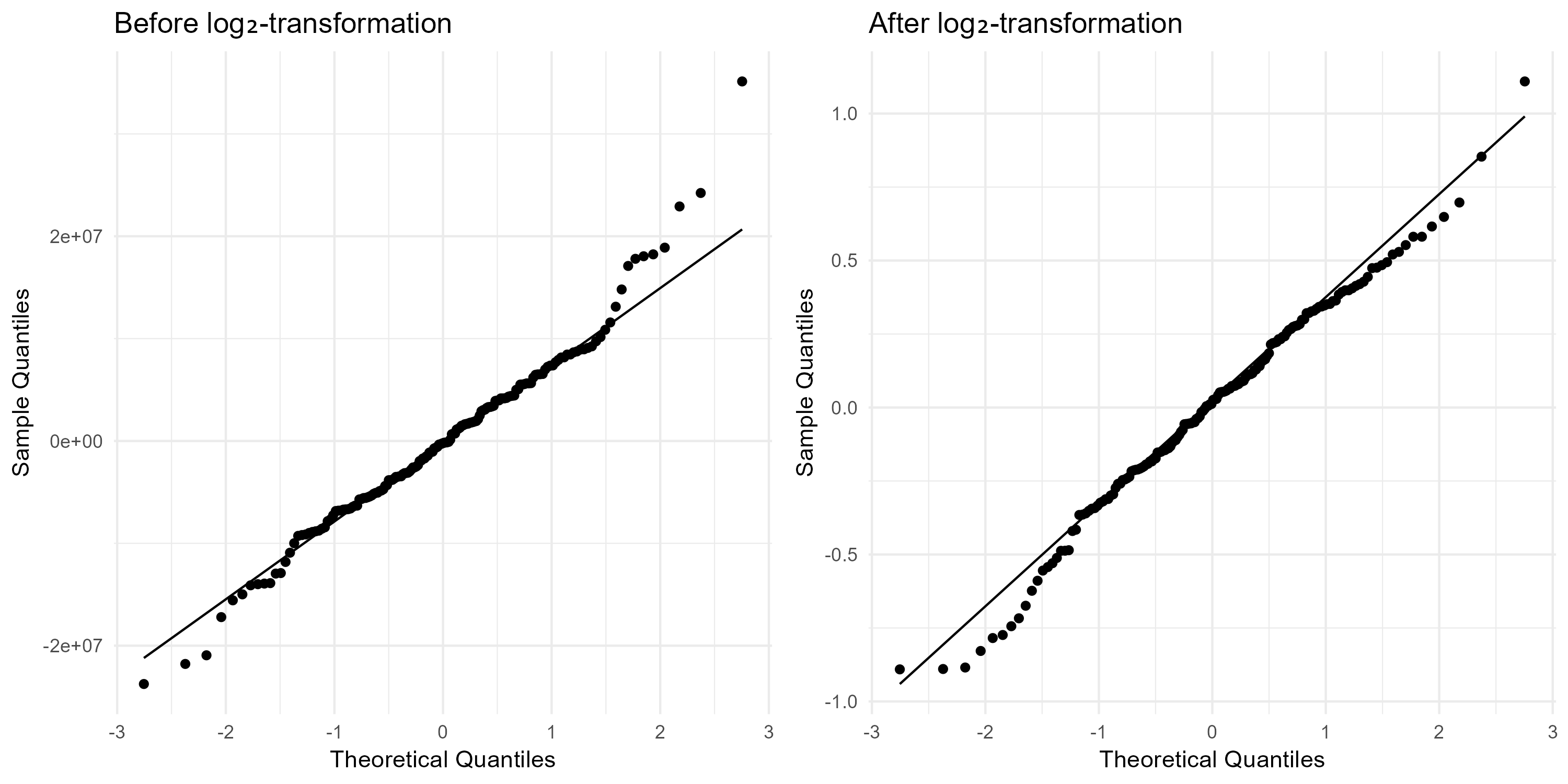


**Supplementary Figure 1** QQ plots of residuals from regression models for 25 randomly chosen metabolites, both before and after the log₂‑transformation

The QQ plots compare the observed quantiles of the sample (y-axis) against the expected (theoretical) quantiles of a normal distribution (x-axis). The purpose of these plots is to assess how closely the data follows a normal distribution. The closer the data points align with the diagonal line, the more closely the residuals follow a normal distribution
