# Supplementary material for: Exercise intensity determines circulating levels of Lac-Phe and other exerkines: a randomized crossover trial
Source: Metabolomics. 2025 May 7;21(3):63. doi: 10.1007/s11306-025-02260-0 (PMC12058925; doi:10.1007/s11306-025-02260-0)
Supplement: Supplementary file 2 — Supplementary file2 (DOCX 966 KB) [file 11306_2025_2260_MOESM2_ESM.docx]

**Exercise intensity determines circulating levels**

**of Lac-Phe and other exerkines:**

**a randomized crossover trial**

Dirk Weber^1^, Paola G. Ferrario^2^, Achim Bub^1,2^

^1^ Institute of Sports and Sports Science, Karlsruhe Institute of Technology, Karlsruhe, Germany,

^2^ Department of Physiology and Biochemistry of Nutrition, Max Rubner-Institute, Karlsruhe, Germany

*Metabolomics (Springer)*

**Corresponding author:**

Dirk Weber

Karlsruhe Institute of Technology (KIT)

Engler-Bunte-Ring 15

76131 Karlsruhe (Germany)

[dirk.weber@kit.edu](mailto:dirk.weber@kit.edu)


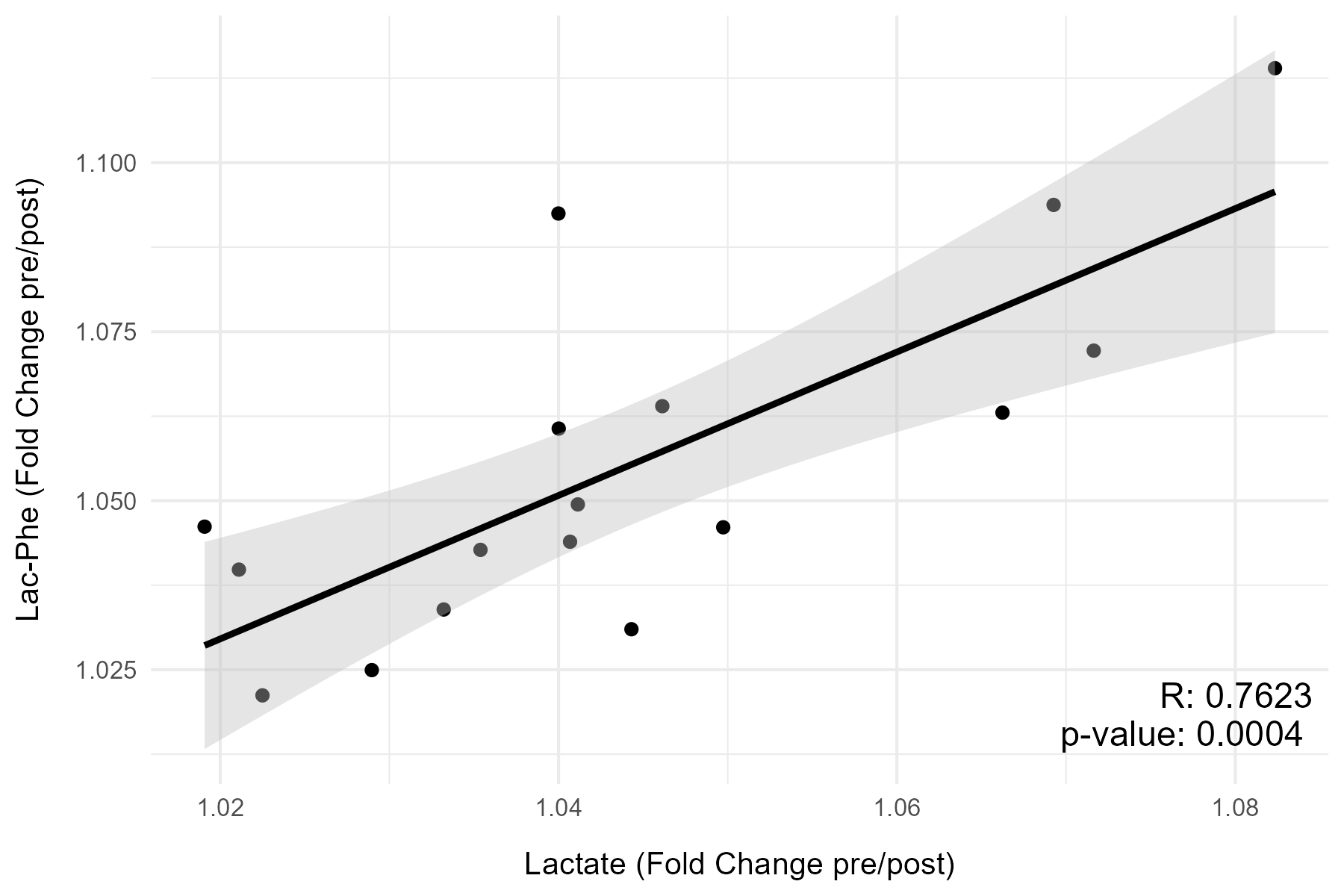


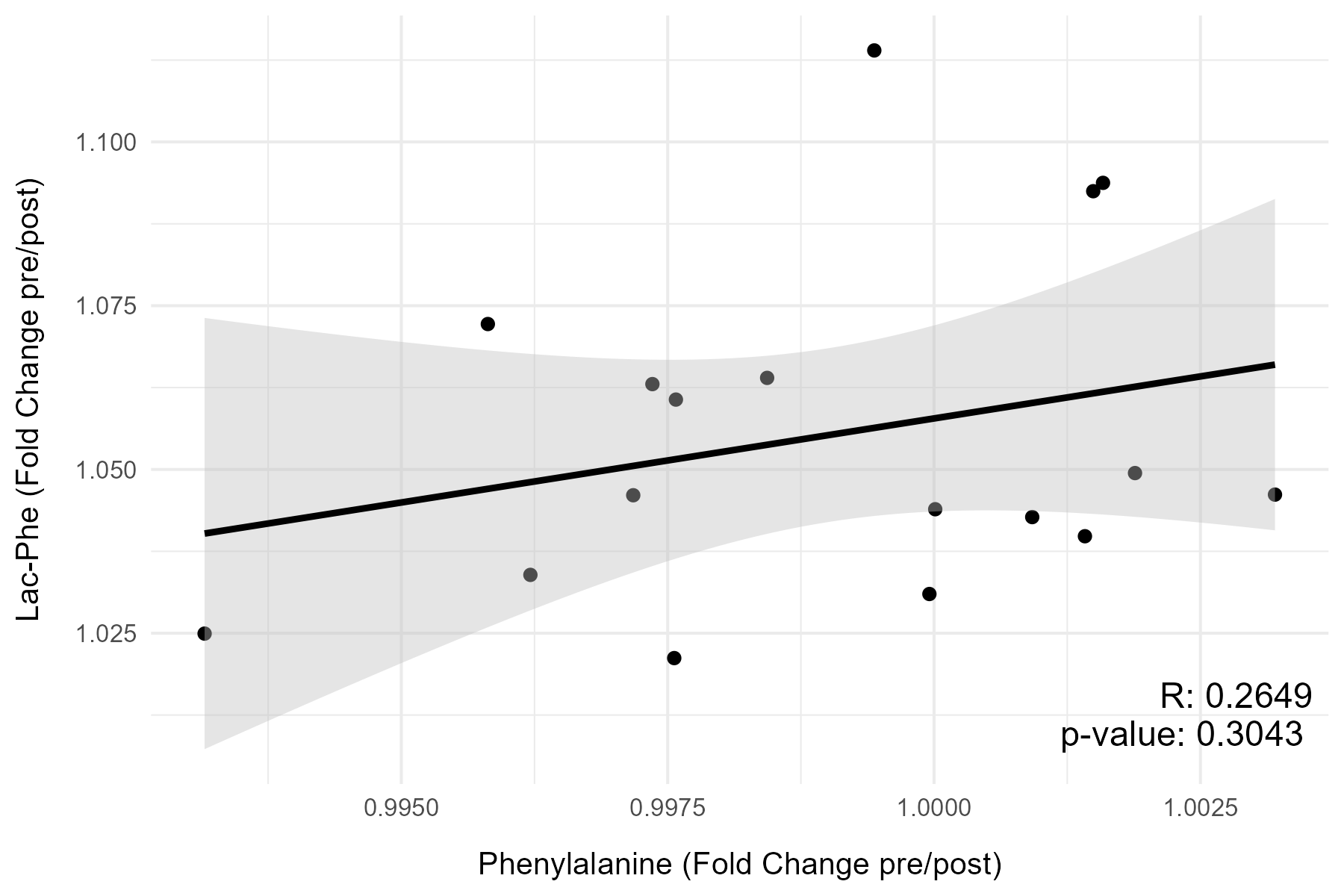


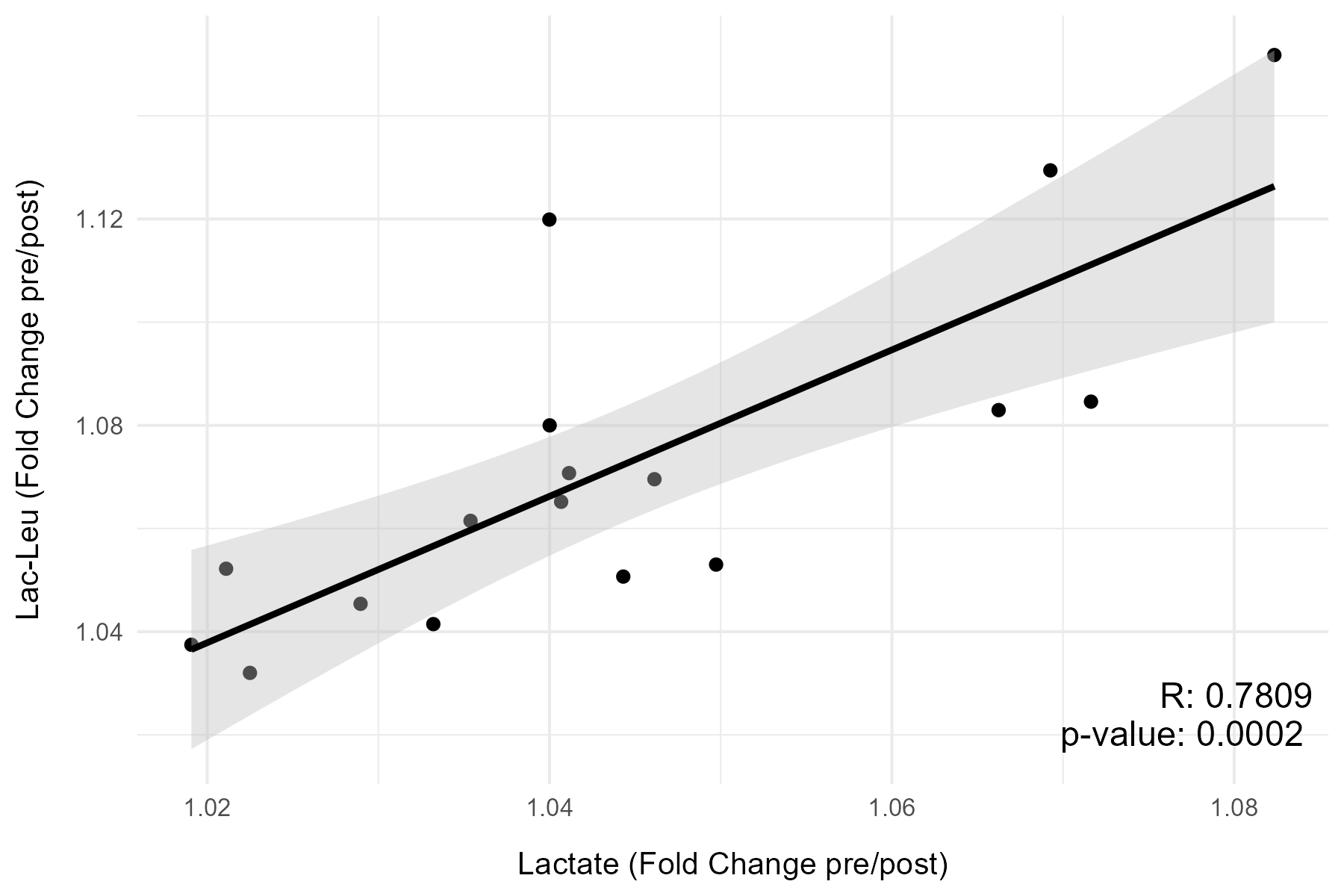


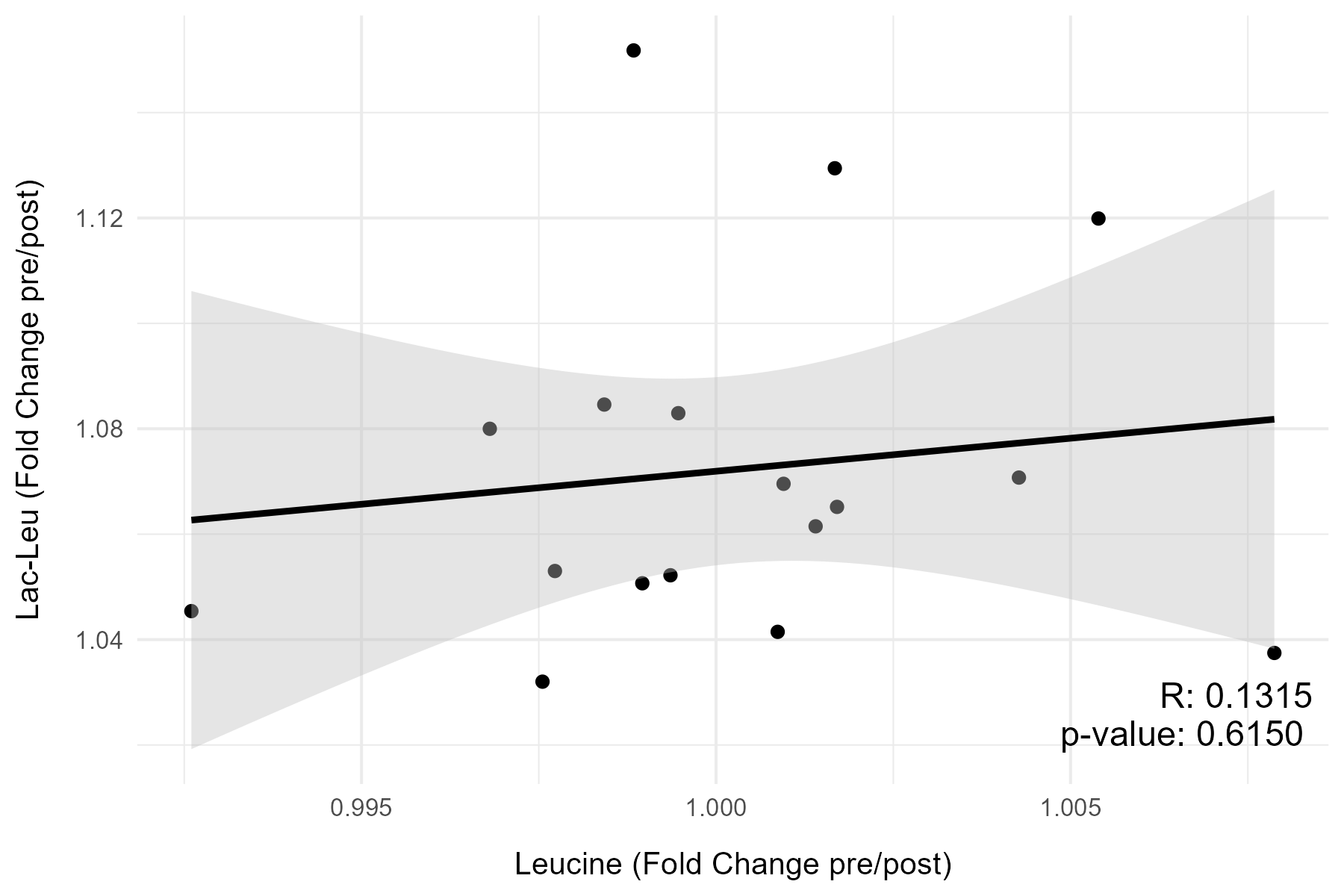


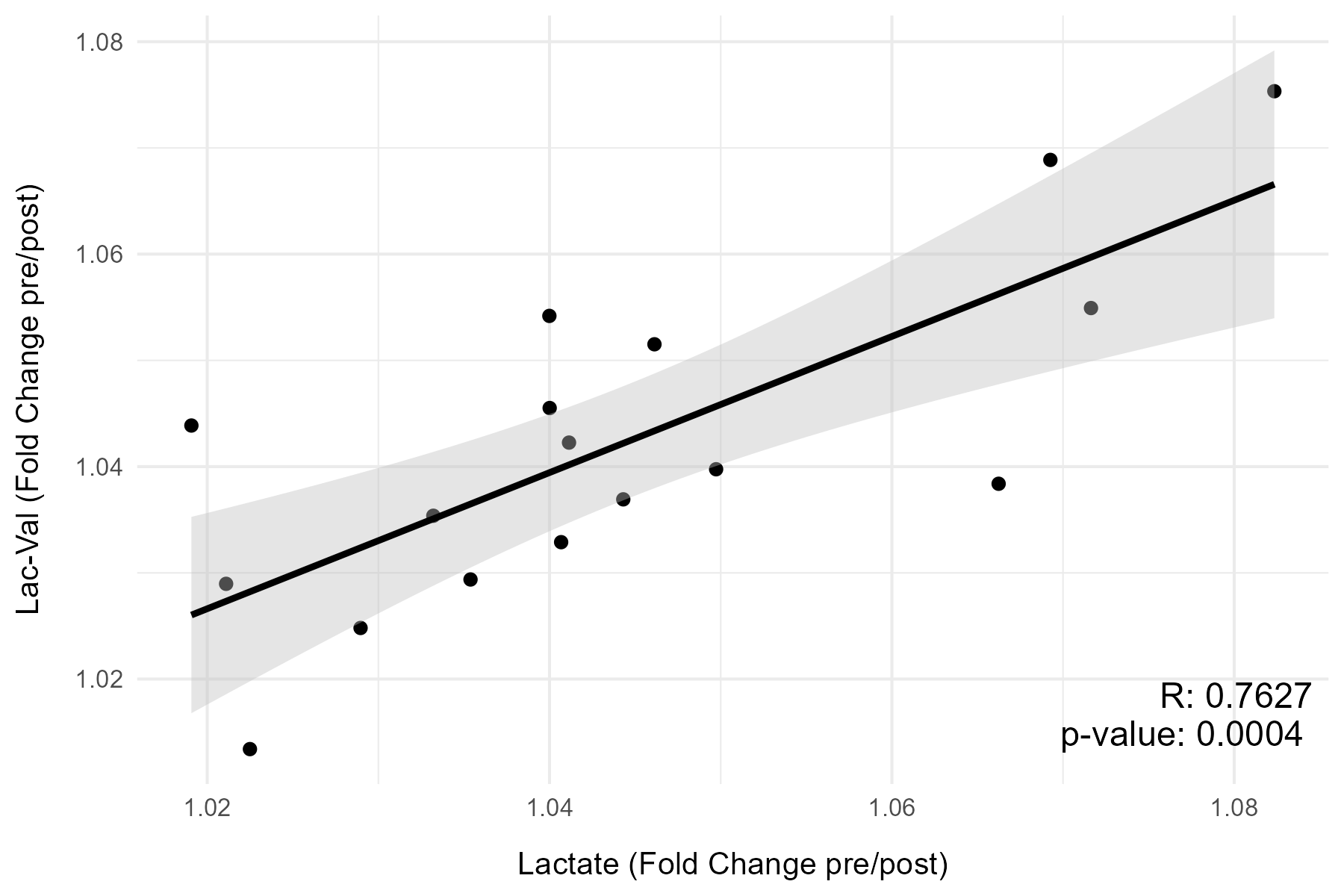


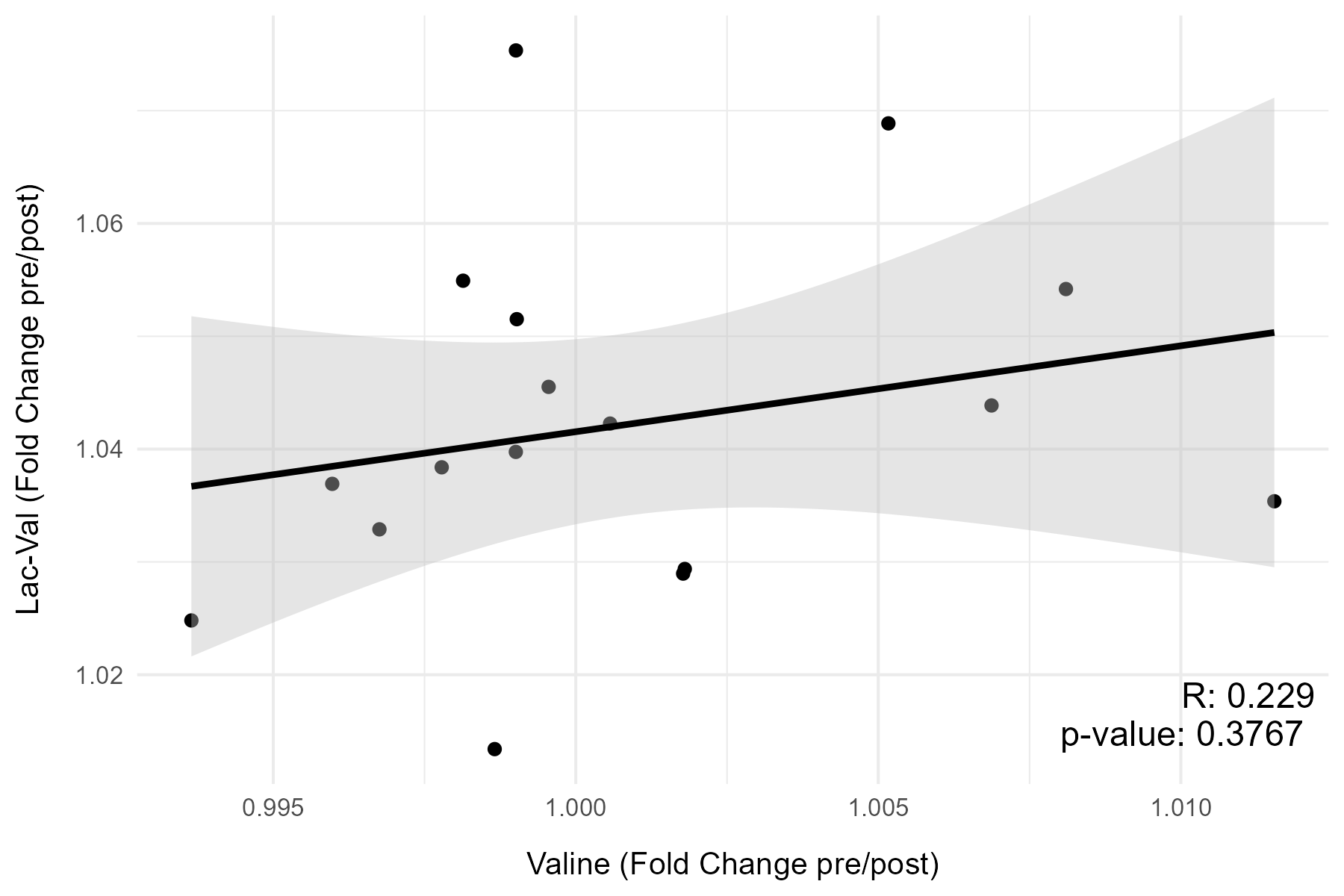


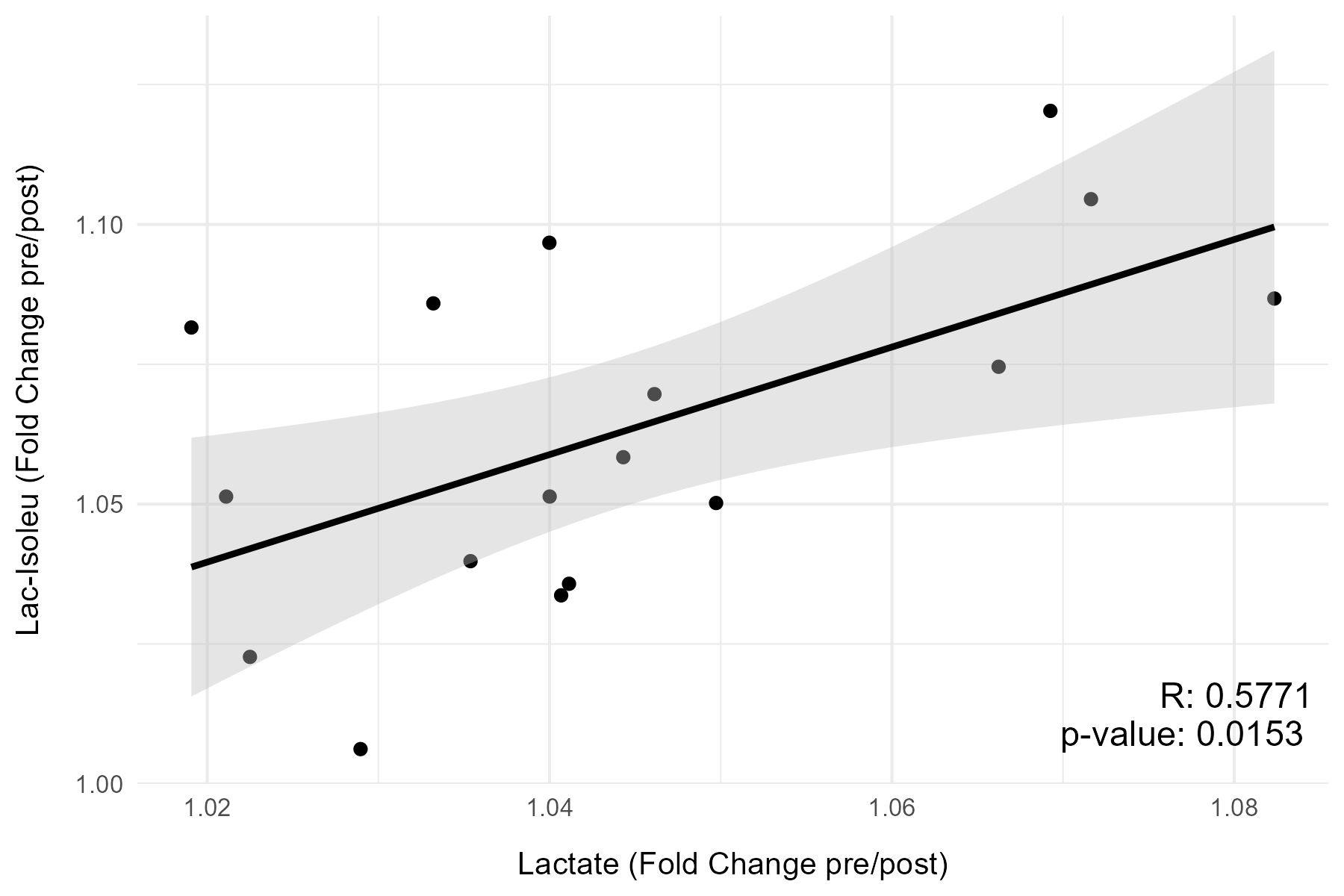


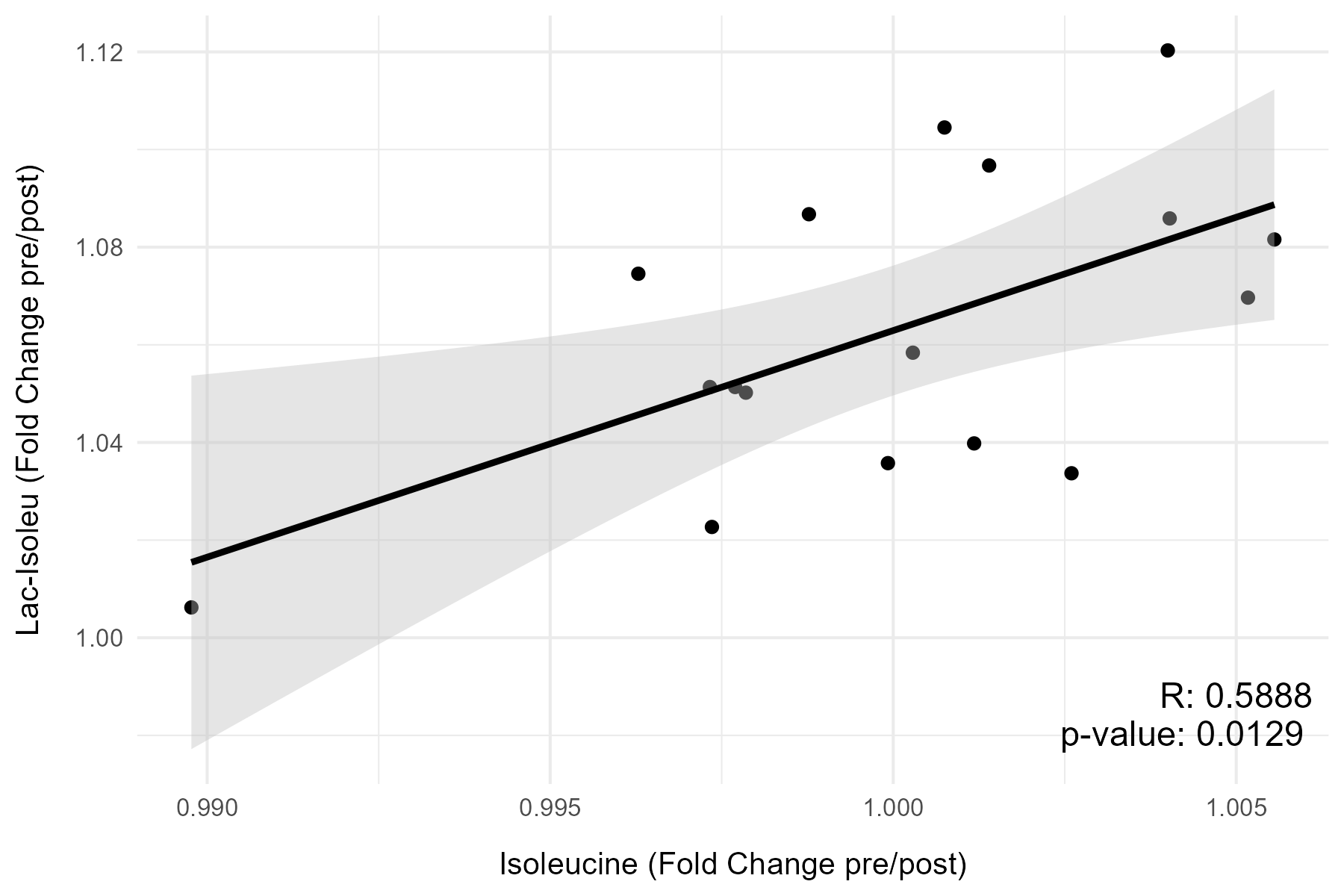


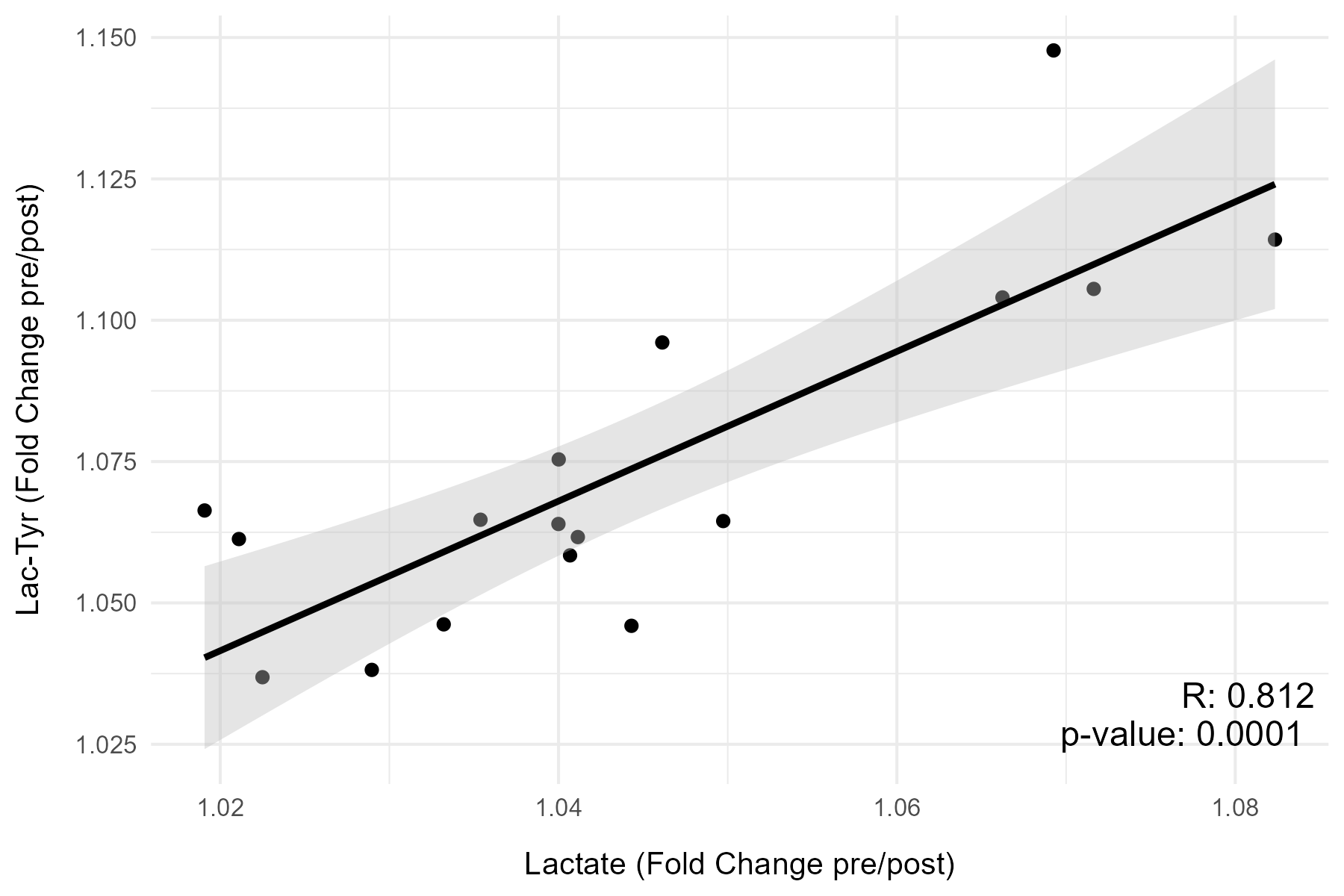


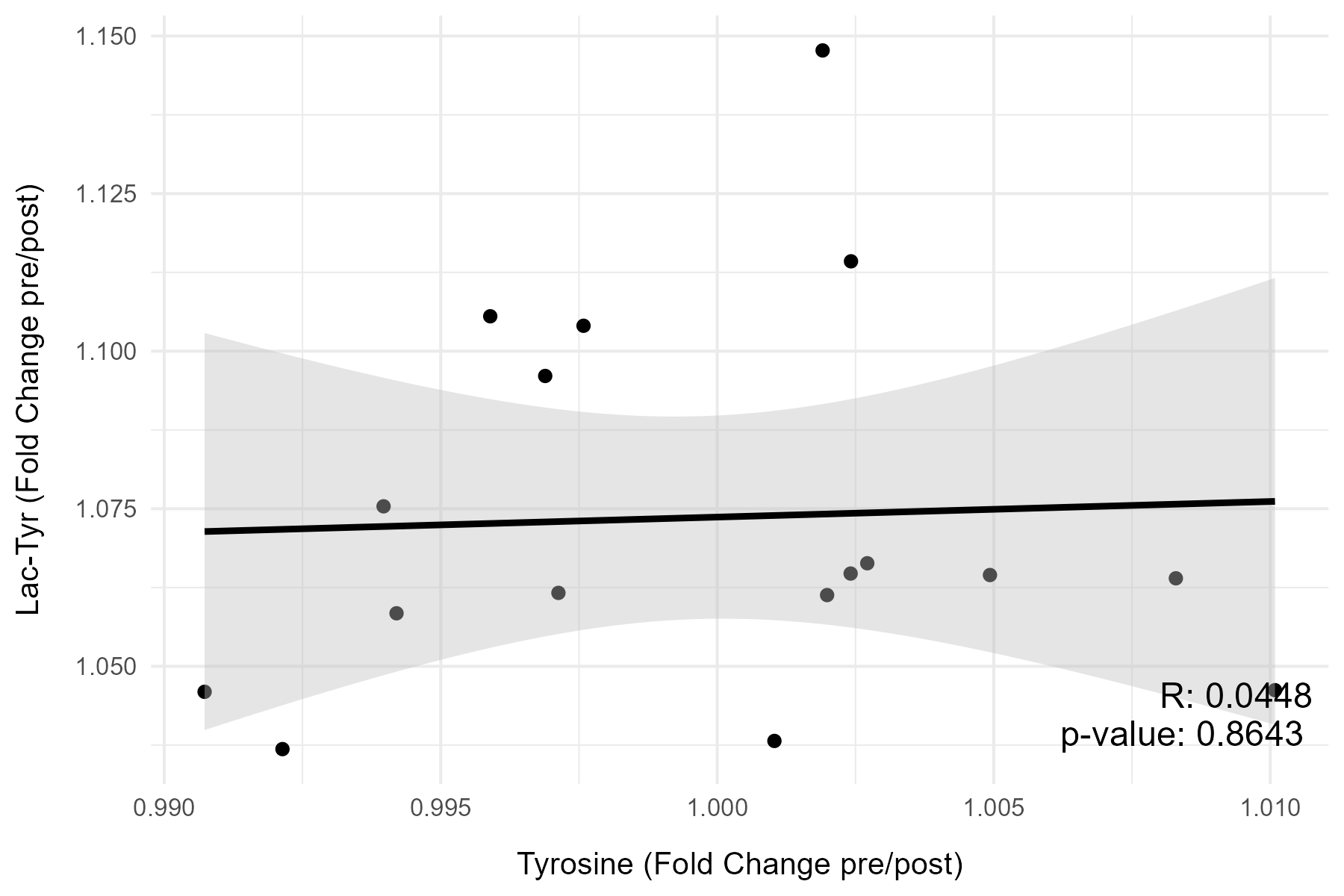


**Supplementary Figure 2** Simple linear regressions between N-lactoyl-amino acids and lactate as well as corresponding amino acids

Data represents the fold change from baseline (t_0_) to immediately post exercise (t_1_) for N-lactoyl-amino acids, lactate and corresponding amino acids
