# Supplementary material for: Exercise intensity determines circulating levels of Lac-Phe and other exerkines: a randomized crossover trial
Source: Metabolomics. 2025 May 7;21(3):63. doi: 10.1007/s11306-025-02260-0 (PMC12058925; doi:10.1007/s11306-025-02260-0)
Supplement: Supplementary file 3 — Supplementary file3 (DOCX 658 KB) [file 11306_2025_2260_MOESM3_ESM.docx]

**Exercise intensity determines circulating levels**

**of Lac-Phe and other exerkines:**

**a randomized crossover trial**

Dirk Weber^1^, Paola G. Ferrario^2^, Achim Bub^1,2^

^1^ Institute of Sports and Sports Science, Karlsruhe Institute of Technology, Karlsruhe, Germany,

^2^ Department of Physiology and Biochemistry of Nutrition, Max Rubner-Institute, Karlsruhe, Germany

*Metabolomics (Springer)*

**Corresponding author:**

Dirk Weber

Karlsruhe Institute of Technology (KIT)

Engler-Bunte-Ring 15

76131 Karlsruhe (Germany)

[dirk.weber@kit.edu](mailto:dirk.weber@kit.edu)

**Supplementary Table 1** Overview of inclusion and exclusion criteria for study participation

| **Inclusion Criteria** | **Exclusion Criteria** |
| --- | --- |
| - Healthy and physically active men - 18 years or older - Non-smokers - Provided written and informed consent | - Smokers - Volunteers with diseases of the gastrointestinal tract, metabolism, nervous system and infectious or immunological diseases in therapeutic need - Volunteers with tumors, acute or chronic infectious diseases - Volunteers with diseases of the cardiovascular system and/or cardiac pacemaker - Institutionalized patients in psychiatric hospitals - Volunteers with intolerances against gluten, fructose or lactose - Volunteers who donated blood in the last three months - Volunteers who may not adhere to the study protocol |
