# Supplementary material for: Exercise intensity determines circulating levels of Lac-Phe and other exerkines: a randomized crossover trial
Source: Metabolomics. 2025 May 7;21(3):63. doi: 10.1007/s11306-025-02260-0 (PMC12058925; doi:10.1007/s11306-025-02260-0)
Supplement: Supplementary file 4 — Supplementary file4 (DOCX 15 KB) [file 11306_2025_2260_MOESM4_ESM.docx]

**Exercise intensity determines circulating levels**

**of Lac-Phe and other exerkines:**

**a randomized crossover trial**

Dirk Weber^1^, Paola G. Ferrario^2^, Achim Bub^1,2^

^1^ Institute of Sports and Sports Science, Karlsruhe Institute of Technology, Karlsruhe, Germany,

^2^ Department of Physiology and Biochemistry of Nutrition, Max Rubner-Institute, Karlsruhe, Germany

*Metabolomics (Springer)*

**Corresponding author:**

Dirk Weber

Karlsruhe Institute of Technology (KIT)

Engler-Bunte-Ring 15

76131 Karlsruhe (Germany)

[dirk.weber@kit.edu](mailto:dirk.weber@kit.edu)

**Supplementary Table 2** Instruments utilized in this study and their corresponding purposes

| **Instrument** | **Purpose** |
| --- | --- |
| Seca 285, Hamburg, Germany | Measurement of participants’ body weight and height |
| BIA Nutriguard MS, Data Input, Pöcking, Germany | Bioelectrical Impedance Analysis (BIA) |
| NutriPlus software (version 5.3.0) | Calculation of lean body mass, fat mass and percent fat mass from the BIA output |
| Boso Carat Professional, Bosch & Sohn, Jungingen, Germany | Measurement of resting heart rate and systolic and diastolic blood pressure |
| SRM Sport High Performance, SRM, Jülich, Germany | Bicycle ergometer |
| Polar H7, Polar, Kempele, Finland | Heart rate sensor during the exercise intervention |
| S-Monovette® tubes (9 mL, K3 EDTA, Sarstedt, Nümbrecht, Germany | Collection of blood samples |
| german-cryo®, Jüchen, Germany | Cryopreserving of samples |
| ggplot2 package in R | Generation of plots |
